# Assessing the genetic relationship between gastroesophageal reflux disease and chronic respiratory diseases: a mendelian randomization study

**DOI:** 10.1186/s12890-023-02502-8

**Published:** 2023-07-04

**Authors:** Xiaoxue Cheng, Jiang Shi, Ding Zhang, Caichen Li, Haoxiang Xu, Jianxing He, Wenhua Liang

**Affiliations:** 1 Nanshan School, Guangzhou Medical University, Guangzhou, 511436 China; 2 Department of Thoracic Surgery and Oncology, The First Affiliated Hospital of Guangzhou Medical University, Guangzhou, 510120 China; 3 State Key Laboratory of Respiratory Disease, Guangzhou, 510120 China; 4 National Clinical Research Center for Respiratory Disease, Guangzhou, 510120 China; 5 Department of Gastroenterology, Maoming Hospital of Guangzhou University of Chinese Medicine, Maoming, China; 6 The Second Affiliated Hospital, Guangdong Provincial Hospital of Chinese Medicine) of Guangzhou University of Chinese Medicine, Guangzhou, 510120 China

**Keywords:** Gastroesophageal reflux disease, Chronic respiratory diseases, Mendelian randomization, Genetic, Causality

## Abstract

**Background:**

Previous observational studies have found an association between gastroesophageal reflux disease (GERD) and chronic respiratory diseases, but it remains uncertain whether GERD causally influences these diseases. In this study, we aimed to estimate the causal associations between GERD and 5 chronic respiratory diseases.

**Methods:**

88 GERD-associated single nucleotide polymorphisms (SNPs) identified by the latest genome-wide association study were included as instrumental variables. Individual-level genetic summary data of participants were obtained from corresponding studies and the FinnGen consortium. We applied the inverse-variance weighted method to estimate the causality between genetically predicted GERD and 5 chronic respiratory diseases. Furthermore, the associations between GERD and common risk factors were investigated, and mediation analyses were conducted using multivariable MR. Various sensitivity analyses were also performed to verify the robustness of the findings.

**Results:**

Our study demonstrated that genetically predicted GERD was causally associated with an increased risk of asthma (OR 1.39, 95%CI 1.25–1.56, *P* < 0.001), idiopathic pulmonary fibrosis (IPF) (OR 1.43, 95%CI 1.05–1.95, *P* = 0.022), chronic obstructive disease (COPD) (OR 1.64, 95%CI 1.41–1.93, *P* < 0.001), chronic bronchitis (OR 1.77, 95%CI 1.15–2.74, *P* = 0.009), while no correlation was observed for bronchiectasis (OR 0.93, 95%CI 0.68–1.27, *P* = 0.645). Additionally, GERD was associated with 12 common risk factors for chronic respiratory diseases. Nevertheless, no significant mediators were discovered.

**Conclusions:**

Our study suggested that GERD was a causal factor in the development of asthma, IPF, COPD and chronic bronchitis, indicating that GERD-associated micro-aspiration of gastric contents process might play a role in the development of pulmonary fibrosis in these diseases.

**Supplementary Information:**

The online version contains supplementary material available at 10.1186/s12890-023-02502-8.

## Background

Gastroesophageal reflux disease (GERD) is a multifaceted disorder with multiple symptoms, such as heartburn, regurgitation, chest or epigastric pain, nausea, bloating, and so on, which occurs when the contents of the stomach reflux into the esophagus or laryngopharynx [1]. The prevalence of GERD is around 15–20% in western countries and has increased from 18.1 to 27.8% in North America, from 8.8 to 25.9% in Europe, and from 2.5 to 7.8% in East Asia 2005 to 2011 [2, 3].

Chronic respiratory diseases, such as idiopathic pulmonary fibrosis (IPF), chronic obstructive disease (COPD), and asthma, share common features of airway inflammation that can be exacerbated by prolonged exposure to gastric fluid, indicating that GERD might contribute to their development [4]. Epidemiological evidence has been accumulating over the past decade linking GERD with an increased risk of chronic respiratory diseases. Several studies have reported that GERD was a significant risk factor for asthma [5, 6], COPD [7], and IPF [8, 9]. For example, compared with population controls, the odds of having GERD were higher in IPF cases [8]. A recent meta-analysis also found an association between GERD and increased risk of IPF, although the relationship was not significant after adjusting for smoking [9].

However, the results of observational studies may be influenced by potential confounders and reverse causality, leading to inconclusive conclusions. For instance, few studies have controlled for body mass index (BMI), which is known to be correlated with the risk of both GERD and chronic respiratory diseases. Additionally, other confounding factors such as smoking status and alcohol consumption may also interfere with the results [10, 11] .Therefore, the existence of a true causal relationship between GERD and chronic respiratory diseases remains uncertain.

Mendelian randomization (MR) analysis is an increasingly popular epidemiological approach for assessing the causation between an exposure and an outcome. By using genetic variants as instrumental variables (IVs), MR analysis is less susceptible to unobserved confounders and reverse causation [12]. Since genetic variations are randomly distributed at conception according to Mendel’s second law, they are generally independent of environmental risk factors and precede the development of diseases [13]. Moreover, with the availability of summary data from genome-wide association studies (GWASs) databases, it is possible to explore the potential relationship and causality between GERD and chronic respiratory diseases. In this study, we utilized published GWASs summary data and conducted MR analyses to comprehensively evaluate the causality [14, 15].

## Materials and methods

**Fig. 1:**
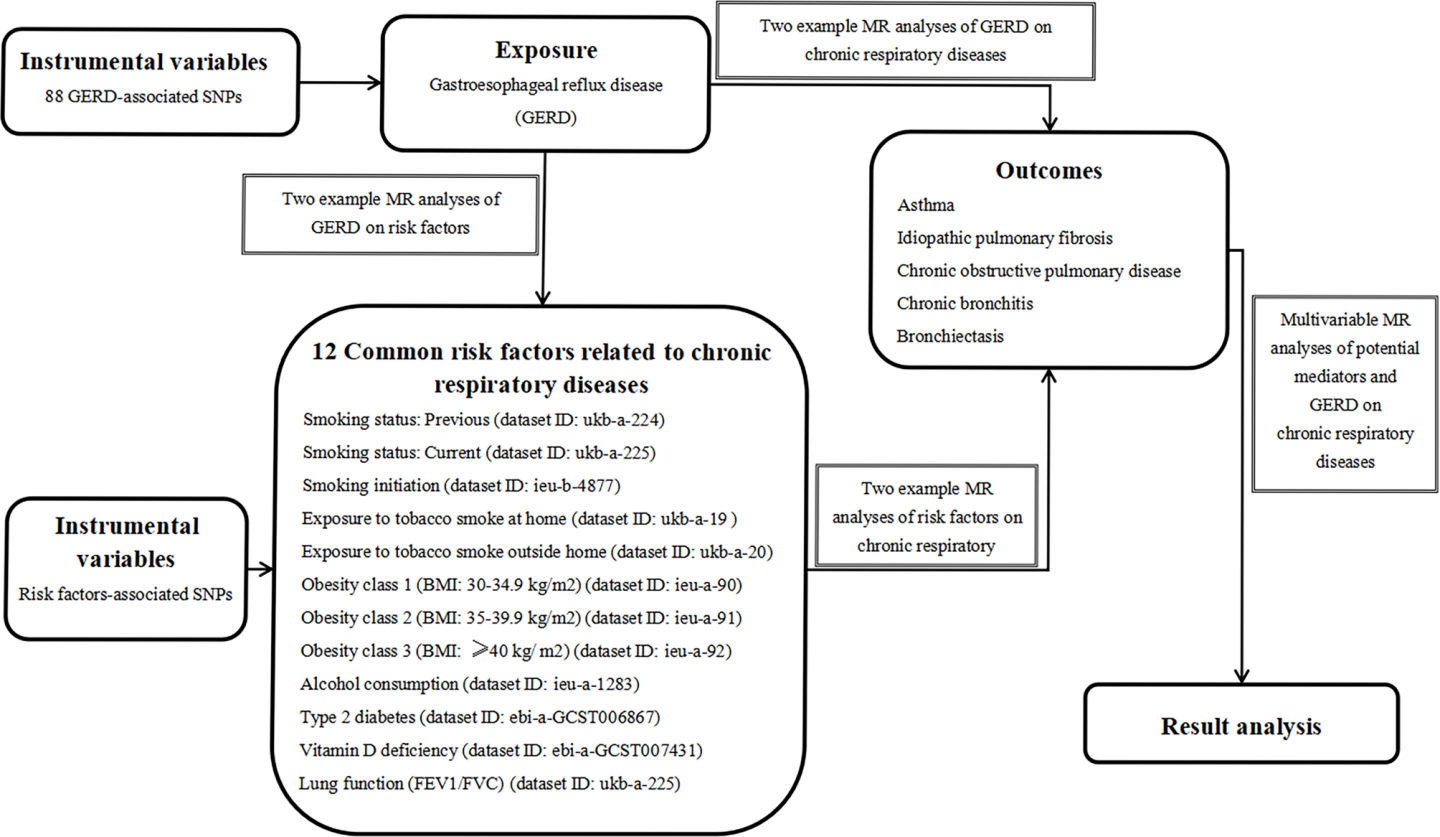
The framework of Mendelian randomization analysis in our study. MR, mendelian randomization; SNPs, single-nucleotide polymorphisms

Two-sample MR analyses were performed to investigate the causal relationship between GERD and the risk of 5 chronic respiratory diseases, as well as 12 related risk factors. In addition, multivariable MR analyses were performed to assess the direct causal impacts of GERD on chronic respiratory diseases by adjusting for each potential mediator, which was statistically significant. Our complete study design overview is depicted in Fig. 1.

## Genetic instruments data for GERD

We obtained IVs for GERD from a meta-analysis of GWASs in European populations, which combined data from the UK Biobank study and the QSKIN study, including a total of 78,707 cases and 288,734 controls. In the meta-analysis, 88 independent lead single-nucleotide polymorphisms (SNPs) that surpassed the genome-wide significance threshold of p < 5 × 10^− 8^ were identified [16]. These IVs collectively explained 3.9% of the variance in GERD [17]. Subsequently, 14 out of the 88 SNPs were removed after conducting linkage disequilibrium (LD) analysis with a threshold of R^2^ < 0.001. Additionally, after harmonizing the genetic data of GERD and chronic respiratory diseases, three SNPs (rs2145318, rs2358016, rs957345) were excluded from the IV set, as they were prone to bias in two-sample MR analysis due to their palindromic nature and intermediate allele frequencies. Ultimately, a total of 71 SNPs were included in the final IV set as genetic variables. Detailed information on the IVs for GERD can be found in Supplementary Data Table S1.

### GWASs summary data on 5 chronic respiratory diseases

Our research aimed to determine whether there is a causal link between GERD and several chronic respiratory diseases that commonly have high morbidity rates and wide-ranging impacts on both males and females. To achieve this, we included five chronic respiratory diseases as outcomes in our primary analyses, namely asthma, COPD, IPF, bronchiectasis, and chronic bronchitis. To ensure the accuracy of our results, we applied strict inclusion criteria, including using publicly available summary data on chronic respiratory diseases released within the past five years and studying patients of European ancestry. Additionally, the diagnostic approach of included patients based not only on ICD-coding, but also other codes such as examinations or drugs. The genetic participants’ sample for asthma was obtained from a large GWAS summary data, which included 56,167 cases and 352,255 controls [18]. Asthma cases from UK Biobank accepted a broad definition based on a diagnosis from UK Biobank Outcome Adjudication Group, hospital, primary care, and self-reported related records (data-field 20,002 in UK Biobank). Additionally, we included the summary data of IPF from the largest available GWASs to date, which was a meta-analysis including 4125 IPF cases and 20,464 controls [19]. IPF cases were identified based on primary or secondary ICD10 code J84.1 (Other interstitial pulmonary diseases with fibrosis). We are grateful to the FinnGen group for their hard work in providing us with summary statistics for COPD (16,410 cases and 283,589 controls), chronic bronchitis (1,035 cases and 283,589 controls), and bronchiectasis (1,967 cases and 283,589 controls) [20]. COPD cases were defined as those with primary or secondary ICD10 code J43 (Emphysema) or J44 (Other chronic obstructive pulmonary disease), and chronic bronchitis cases were defined as those with primary or secondary ICD10 code J42 (Unspecified chronic bronchitis) [20]. Detailed information on studies included in analyses is presented in Table 1. Additionally, we retrieved data from other groups in the UK Biobank and FinnGen group to replicate the casual association between genetically predicted GERD and chronic respiratory diseases (Supplementary Data Table S10 and S11).

**Table 1:** Details of studies included in Mendelian randomization analyses

Trait	First author	Consortium	Number of cases	Number of controls	PubmedID	Population	Year
Asthma	Valette K	NA	56,167	352,255	34,103,634	European	2021
Idiopathic pulmonary fibrosis	Allen RJ	NA	4125	20,464	35,688,625	European	2022
Chronic obstructive pulmonary disease	NA	FinnGen	16,410	283,589	NA	European	2022
Chronic bronchitis	NA	FinnGen	1035	283,589	NA	European	2022
Bronchiectasis	NA	FinnGen	1967	283,589	NA	European	2022

### GWASs summary data on 12 common risk factors of chronic respiratory diseases

We collected summary data for 12 common risk factors associated with chronic respiratory diseases. These included smoking status (previous and current) from Neale Lab (dataset ID: ukb-a-224 and ukb-a-225) [21], initiating smoking from the GWAS & Sequencing Consortium of Alcohol and Nicotine use (GSCAN; dataset ID: ieu-b-4877) [22], exposure to tobacco smoke at home and outside home from Neale Lab (dataset ID: ukb-a-19 and ukb-a-20), obesity class 1–3 from the Genetic Investigation of ANthropometric Traits (GIANT; dataset ID: ieu-a-90, ieu-a-91, ieu-a-92) [23], alcohol consumption from the UK Biobank (dataset ID: ieu-a-1283) [24], Type 2 diabetes from the open GWAS study (dataset ID: ebi-a-GCST006867) [25], Vitamin D deficiency from the FinnGen group (dataset ID: finn-b-E4_VIT_D_DEF) [20], and lung function (FEV1/FVC) from the open GWAS study (dataset ID: ebi-a-GCST007431) [26]. Detailed information on the studies mentioned above is presented in Supplementary Data Table S2.

### Statistical analysis

#### Two-sample MR analyses between GERD and risk of 5 chronic respiratory diseases outcomes

We first applied a two-sample MR model to access the causal effect of GERD on 5 chronic respiratory diseases. The main MR analyses were performed using the inverse-variance weighted (IVW) method, which involves a meta-analysis of the Wald ratio of each single nucleotide polymorphism (SNP) between the exposure (GERD) and the outcome (chronic respiratory diseases) using a random-effects inverse-variance method. This approach weights each ratio based on its standard error, taking into account possible heterogeneity [27]. In this regard, given that phenotype of GERD (X) on the risk of outcomes (Y) and the genetic variants (G) as IVs, the IVW analyses were performed by combining genetic variants on GERD (bYG) with their standard errors (SEYG) to examine the causal relationship between genetically determined exposure factor (β_GERD_) and outcome phenotypes. Odds ratios (ORs) with 95% confidence intervals (CIs) were calculated and estimated for the five chronic respiratory diseases. We considered a causal relationship as significant if the Bonferroni-corrected threshold P-value was < 0.01 (0.05/5), indicating a stringent correction for multiple testing.

#### Two-sample MR analyses the associations of GERD and 12 common risk factors for chronic respiratory diseases

We further conducted Mendelian randomization (MR) analysis to investigate the potential pathogenesis from gastroesophageal reflux disease (GERD) to chronic respiratory diseases by examining whether genetic susceptibility for GERD could be associated with common risk factors of chronic respiratory diseases. Although the underlying pathophysiological processes of many of them has not been fully understood, previous studies have observed associations between potential genetic and environmental factors and exacerbation risk of these diseases [28–32]. In our analysis, we used the inverse-variance weighted (IVW) method to estimate the causal effects of genetically predicted GERD on 12 common risk factors for chronic respiratory diseases. We applied a Bonferroni correction with a significance threshold of P-value < 4.2 × 10^− 3^ (0.05/12) to account for multiple testing.

#### Two-sample MR analyses between genetically predicted risk factors and risk of five chronic respiratory diseases outcomes

Furthermore, we selected specific risk factors that showed a significant causal relationship with GERD to estimate their causal impact on individual chronic respiratory diseases, for which GERD was statistically significant. We obtained IVs that were independent genome-wide significant (R^2^ < 0.001) for smoking initiation, obesity class 1–3, Alcohol consumption, Type 2 diabetes, Vitamin D deficiency, and Lung function (FEV1/FVC) from the summary data of corresponding GWASs [20, 22–26]. IVs for smoking status, exposure to tobacco smoke, and vitamin D deficiency were obtained from the MR-base database (https://gwas.mrcieu.ac.uk/) (accessed on 25 December 2022) [21]. Additionally, we applied a Bonferroni-corrected P-value < 1.3 × 10^− 2^ (0.05/4; 4 outcomes that GERD was significantly associated with) as the significance threshold.

Assuming that causal relationships were observed in all three previously mentioned analyses, the risk factor being analyzed was considered as a potential pathway mediator linking GERD to this particular chronic respiratory disease outcome.

### Multivariable MR analysis and mediation analysis

We further performed multivariable MR analysis to identify potential mediators between GERD and chronic respiratory diseases and investigate their influence on causal estimates. Multivariable MR analysis c allows for a more accurate estimation of the true causal effect by evaluating the effects of multiple genetic variations and multiple confounding factors in the causal relationship [33]. Specifically, multivariable MR analysis can simultaneously consider the effects of multiple sets of genetic variations and use multiple linear regression to control multiple confounding factors to eliminate their effects on the causal relationship. Multivariable MR analysis can also compare the causal effects between different genetic variations and outcome variables to further address potential collinearity and reverse causality problems. Through these methods, multivariable MR analysis can more accurately test causal hypotheses without the need for complex statistical methods and mathematical models [33]. If the analysis reveals that a specific risk factor significantly weakened the causal association between GERD and diseases of outcomes (P < 0.01 changed to P > 0.05), the risk factor could be identified as a mediator [33].

### Sensitivity analysis

We took advantage of the online web tool (https://shiny.cnsgenomics.com/mRnd/) (accessed on 30 December 2022) to estimate the F statistics and power for the MR analysis [34]. In addition, we employed several other methods, including the Mendelian Randomization Pleiotropy Residual Sum and Outlier (MR-PRESSO) test, MR-Egger regression, and weighted-median estimator, to comprehensively evaluate our MR results [35]. Among them, the MR-PRESSO global test could detect the horizontal pleiotropy of overall IVs based on a regression framework. Moreover, its outlier test can produce a corrected estimate by performing outlier removal if desired, and the distortion test could compare whether there was a significant difference between the results before and after outlier correction [36]. Furthermore, MR-Egger regression, which assumed independent pleiotropic associations, was conducted to explore pleiotropy and estimate causality adjusted for pleiotropy [37]. We also conducted leave-one-out analyses to assess the potential bias from single sensitive SNPs in the IVW method. Additionally, we performed the MR-heterogeneity test using Cochran’s Q test on the IVW and MR-Egger estimate to identify SNPs contributing to heterogeneity in causal estimation [38].

All of our MR analyses were performed in R (version 4.2.1) using the package TwoSampleMR (version 0.5.6) and MRPRESSO (version 1).

## Results

In our study, all 71 SNPs that we selected were chosen based on the genome-wide significance threshold of p < 5 × 10^− 8^, ensuring that they met the MR assumption. The F-statistics for the instrumental variables (IVs) were all above 10, indicating that we did not use weak genetic instruments in our study. Furthermore, the power was sufficient in all MR analyses, except for bronchiectasis, as shown in Supplementary Data Table S3.

### Genetically predicted GERD and 5 chronic respiratory diseases

**Fig. 2:**
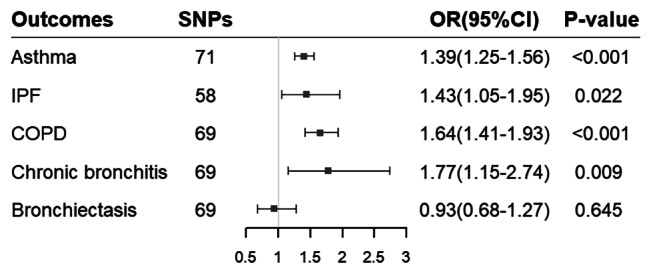
Results that derived from the inverse-variance weighted method displayed the associations between gastroesophageal reflux disease and 5 chronic respiratory diseases. SNPs, single-nucleotide polymorphisms; OR, odds ratio; CI, confidential interval; IPF, idiopathic pulmonary fibrosis; COPD, chronic obstructive pulmonary disease

The result of the IVW method showed a positive association between genetically predicted GERD and asthma (OR 1.39, 95%CI 1.25–1.56, P < 0.001), COPD (OR 1.64, 95%CI 1.41–1.93, P < 0.001) and chronic bronchitis (OR 1.77, 95%CI 1.15–2.74, P = 0.009). Additionally, our study indicated a potential association between genetically predicted GERD and IPF (OR 1.43, 95%CI 1.05–1.95, P = 0.022). However, no significant association was observed between GERD and bronchiectasis (OR 0.93, 95%CI 0.68–1.27, P = 0.645) (Fig. 2). These results were consistent with MR-PRESSO analysis after excluding this outlier. Causal estimates for all five outcomes remained consistent in sensitivity analyses using the weighted median method. Nevertheless, we found that the result of the weighted median method and MR-Egger were not corresponding with them, while they offered less accurate estimates than conventional MR (IVW method). Detailed results of MR using MR-PRESSO, weighted median and MR-Egger method are presented in Table 2 and Supplementary Data Table S4.

**Table 2:** Estimates of sensitivity analyses of GERD and 5 chronic respiratory diseases

Outcomes	MR-PRESSO				Weighted median method			MR-Egger		
	OR (95%CI)	P-value	Outlier number		OR (95%CI)	P-value		OR (95%CI)	P-value	P_intercept_
Asthma	1.46(1.33–1.59)^*^	< 0.001^*^	2		1.36(1.23–1.51)	< 0.001		0.91(0.62–1.33)	0.63	0.026
IPF	1.50(1.14–1.99)	0.01	0		1.27(0.84–1.93)	0.25		4.40(1.57–12.34)	0.01	0.030
COPD	1.62(1.41–1.87)^*^	< 0.001^*^	1		1.44(1.20–1.73)	< 0.001		0.94(0.55–1.61)	0.81	0.036
Chronic bronchitis	1.83(1.25–2.67)	0.003	0		1.84(0.97–3.50)	0.06		2.94(0.63–13.65)	0.17	0.504
Bronchiectasis	0.96(0.73–1.28)	0.80	0		0.85(0.54–1.34)	0.48		0.86(0.28–2.61)	0.78	0.881

IPF, idiopathic pulmonary fibrosis; COPD, chronic obstructive pulmonary disease; *, the results were derived from MR-PRESSO after corrected outliers

### Genetically predicted GERD and risk factors of chronic respiratory diseases

**Fig. 3:**
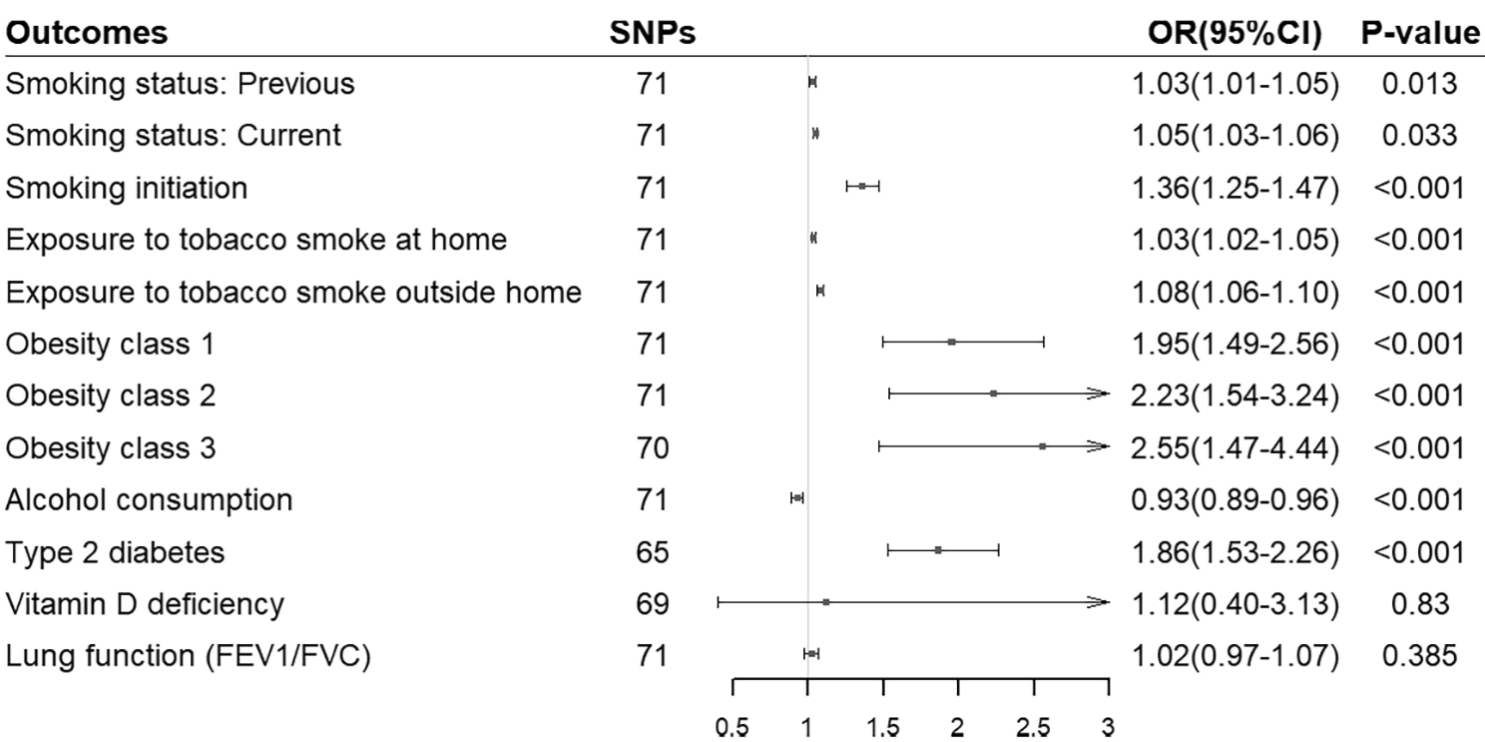
Mendelian randomization associations between genetically predicted gastroesophageal reflux disease and 12 potential risk factors. Obesity class 1, BMI: 30-34.9 kg/m^2^; Obesity class 2, BMI: 35-39.9 kg/m^2^; Obesity class 3, BMI: ≥ 40 kg/ m^2^

GERD were found to be positively associated with seven risk factors, including smoking initiation (OR 1.36, 95%CI 1.25–1.47, P < 0.001), exposure to tobacco smoke at home and outside home (OR 1.03, 95%CI 1.02–1.05, P < 0.001; and OR 1.08, 95%CI 1.06–1.10, P < 0.001 respectively), obesity class 1–3(OR 1.95, 95%CI 1.49–2.56, P < 0.001, OR 2.23, 95%CI 1.54–3.24, P < 0.001, and OR 2.55, 95%CI 1.47–4.44, P < 0.001, respectively), Type 2 diabetes (OR 1.86, 95%CI 1.53–2.26, P < 0.001), and negatively associated alcohol consumption (OR 0.93, 95%CI 0.89–0.96, P < 0.001). Additionally, evidence from the IVW method indicated potential associations between genetically predicted GERD and smoking status (previous and current; OR 1.03, 95%CI 1.01–1.05, P = 0.013, and OR 1.05, 95%CI 1.03–1.06, P = 0.033, respectively). Notably, our analyses showed no statistically significant associations between genetically predicted GERD and vitamin D deficiency (OR 1.12, 95%CI 0.40–3.13, P = 0.830) and lung function (FEV1/FVC; OR 1.02, 95%CI 0.97–1.07, P = 0.385). The MR results using the IVW model are presented in Fig. 3.

### Genetically predicted risk factors and chronic respiratory diseases of outcomes

Further evaluation was conducted to determine whether smoking initiation, exposure to tobacco smoke at home and outside the home, obesity class 1–3, Type 2 diabetes, alcohol consumption, and smoking status (previous and current) were causally connected with four chronic respiratory diseases that GERD was positively associated with. The IVW method indicated that obesity class 1–2 (OR 1.06, 95%CI 1.03–1.09, P < 0.001, and OR 1.05, 95%CI 1.02–1.08, P < 0.001 respectively) and smoking initiation (OR 1.16, 95%CI 1.05–1.28, P = 0.003) were associated with an increased risk for asthma (Supplementary Data Table S5). Additionally, positive associations between smoking initiation (OR 1.72, 95%CI 1.48-2.00, P < 0.001), exposure to tobacco smoke outside home (OR 5.92, 95%CI 1.76–19.93, P = 0.004), current smoking status (OR 135.63, 95%CI 5.93-3100.91, P = 0.002) and COPD were found (Supplementary Data Table S5).

### Multivariable MR analysis

We conducted further analysis to investigate the influence of potential mediators on the causal effects of GERD on asthma and COPD. The results from multivariable MR analyses showed that the causal estimates of GERD with outcomes remained robust even after adjusting for each potential mediator (Table 3).

**Table 3:** Multivariable Mendelian randomization analyses of GERD with common risk factors for chronic respiratory diseases adjusting for potential mediators

Outcomes	Model	OR (95%CI)	P-value
Asthma	Unadjusted model	1.39(1.25–1.56)	< 0.001
	Adjusted for Obesity (Class 1 and 2)	1.33(1.22–1.46)	< 0.001
	Adjusted for Smoking initiation	1.41(1.24–1.62)	< 0.001
COPD	Unadjusted model	1.64(1.41–1.93)	< 0.001
	Adjusted for Smoking initiation	1.27(1.10–1.47)	0.001
	Adjusted for Smoking status (Current)	1.16(1.01–1.34)	0.030
	Adjusted for Exposure to tobacco smoke outside home	1.67(1.40-2.00)	< 0.001

COPD, chronic obstructive pulmonary disease; Obesity class 1, BMI: 30-34.9 kg/m^2^; Obesity class 2, BMI: 35-39.9 kg/m^2^

### Sensitivity analyses

We also performed sensitivity analyses to assess the robustness of our findings. Egger intercept analyses showed little evidence of pleiotropy for asthma, IPF, and COPD, with P-values ranging from > 0.01 to < 0.05 (Table 2 and Supplementary Data Table S6). MR-PRESSO tests identified two outliers for asthma and one outlier for COPD, but no significant statistical evidence of distortion was found through the distortion tests (Table 2 and Supplementary Data Table S7). Overall, we concluded from these analyses that horizontal pleiotropy has little or no interference with our causal estimates. Cochran Q statistics indicated the presence of heterogeneity in the correlation between asthma and COPD (Table 2 and Supplementary Data Table S8). Additionally, no single SNP strongly drove the overall effect of GERD on the five chronic respiratory diseases, as indicated by the leave-one-out sensitivity analysis (Supplementary Figure S1-S5).

## Discussion

With the two-sample MR approach, our study illustrated that patients with GERD were causally associated with an increased risk of asthma, IPF, COPD, and chronic bronchitis, suggesting the process of GERD-associated micro-aspiration of gastric contents may play a role in the development of these respiratory diseases. However, no significant association was observed between GERD and bronchiectasis.

Our results were consistent with previous epidemiological evidence that has reported an increased prevalence of GERD in chronic respiratory diseases. A systematic review in 2010 found that asthma patients were more likely to have GERD compared to controls, with an average prevalence of 22.0% in asthma patients versus 4.8% in controls (pooled OR 5.6, 95% confidence interval [CI] 4.3–6.9) [6]. A recent systematic review and meta-analysis by Mallah et al. also demonstrated an association between GERD and asthma exacerbation (OR 1.27, 95% CI 1.18–1.35) in various study designs and populations, including European and non-European populations [5]. Furthermore, a prospective study found that 21% of patients with severe uncontrolled asthma had signs of GERD detected on bronchoscopy, suggesting that GERD may be a trigger for uncontrolled asthma [39]. The association between GERD and IPF has also been reported in previous studies. Tobin et al. demonstrated a high correlation between abnormal esophageal acid exposure and the incidence of IPF in a comparative study conducted 20 years ago [40]. Recent observational studies have also reported similar findings. A retrospective study by Lee et al. with a sample of 786 IPF patients suggested that the prevalence of GERD increased over the follow-up period, with eventually 107 patients (13.6%) being diagnosed with GERD [41]. A population-based, case-control study also found a higher incidence of GERD in IPF cases compared to population controls (OR 1.78, 95% CI 1.09–2.91, P = 0.02) [8]. In COPD, the prevalence of GERD has been reported to be high, and its association with adverse outcomes has been demonstrated in several studies. A Korean national cross-sectional cohort study found a prevalence of GERD of 28% in COPD patients, and the presence of GERD was associated with an increased risk of hospitalization (OR 1.54, 95% CI 1.50–1.58, P < 0.001) and frequent emergency room visits (OR 1.55, 95% CI 1.48–1.62, P < 0.001) [42]. Similar findings were reported by Huang et al. in a systematic review with meta-analysis, which showed an increased risk of COPD exacerbation associated with GERD (OR 5.37, 95% CI 2.71–10.64) [7]. Another study based on the COPDGene cohort reported a prevalence of GERD of 29.1% in COPD patients, who were more likely to have symptoms of chronic bronchitis and worse scores of life quality [43].

Notably, our MR analysis did not identify a causal relationship between bronchiectasis and GERD, which was somewhat different from previous studies. A recent meta study mentioned that the prevalence of GERD in bronchiectasis ranged from 34 to 74% according to self-reported symptoms and questionnaires and pepsin was detected in sputum samples in 26-70% of patients with mild to moderate bronchiectasis [44]. However, the relationship between bronchiectasis and GORD remains controversial actually. For example, a retrospective cohort study including 192 patients suggested that gastroesophageal reflux was not found to be an important risk factor for the development of bronchiectasis [45]. In addition, two case-control studies did not identify any association with decreased lung function or other markers of disease severity of bronchiectasis patients with GERD. Nevertheless, due to recruitment difficulties, the efficacy of these studies to detect such effects was clearly inadequate and a single time dimension may not be sufficient to accurately reflect the relationship between GERD and bronchiectasis [46, 47]. Consequently, more observational studies are warranted to support our results and investigate their potential connections.

Nevertheless, owing to the flaws of observational research design, there may be some heterogeneity in patient characteristics among the primary studies that could bias the results. For example, GERD could be defined from varied perspectives. According to the Montreal definition, GERD is a condition that develops when the reflux of stomach contents causes troublesome symptoms and/or complications [48]. However, some studies diagnosed GERD based solely on pH-metry, others on a symptom inventory, and only a few inferred from diagnostic code entries, which could generate multiple deviations. Therefore, the genuine relationship between GERD and them might also be biased owing to the inaccurate diagnosis of GERD. Additionally, the association estimates from case-control studies, as indicated by Bédard et al., may have little confidence [9]. Unfortunately, there is still no prospective large-scale longitudinal cohort study that has investigated the relationship between GERD and asthma, idiopathic pulmonary fibrosis (IPF), chronic obstructive pulmonary disease (COPD), bronchiectasis, and chronic bronchitis. The results of our MR analysis revealed that genetically predicted GERD might be associated with an increased risk of asthma, IPF, COPD, and chronic bronchitis, supporting a causal role of GERD in the development of them.

Current research findings have identified several risk factors associated with chronic respiratory diseases, such as Smoking, which is one of the most widely known risk factors for respiratory diseases. Additionally, a growing number of studies have suggested that tobacco smoke exposure may also contribute to the development of asthma, COPD, and chronic bronchitis [30–32]. A 2016 international report indicated that low FVC could be a potential risk factor for acute exacerbation of IPF [28]. Furthermore, higher body mass index and alcohol might also contribute to the exacerbation of IPF, COPD and chronic bronchitis [28, 31, 32]. Interestingly, our further detailed MR analysis confirmed that GERD may be causally associated with a broad range of them, including smoking, smoking status, exposure to tobacco, obesity, alcohol consumption, and type 2 diabetes, while no statistically significant associations were detected with vitamin D deficiency and lung function. Additionally, although smoking is a well-known risk factor for IPF or chronic bronchitis, it did not show statistical significance in this study, other two MR studies have reported similar results. One of them discussed the relationship between hypothyroidism and IPF, and the authors also did not find any statistically significant evidence for an association between smoking and IPF in the multivariable MR analysis based on BMA [49]. Another article explored the role of gastro-esophageal reflux disease in the development of IPF also discovered the same results in the MR analysis using identical data source [50]. Consequently, while smoking may be a risk factor for the development of IPF or chronic bronchitis from a macro epidemiological perspective, it may not be genetically associated with these conditions. Therefore, further GWAS studies with more precise assessment of smoking behavior are warranted. Furthermore, our study also revealed the causal relationship between obesity and asthma, smoking and asthma, tobacco smoke exposure outside home and COPD, smoking and COPD. Therefore, smoking and obesity may act as mediators between GERD and chronic respiratory diseases. After using MVMR analyses adjusted by smoking and obesity, none of the potential confounders mentioned above were statistically significant mediators and the direct causal estimates of genetically predicted GERD with the outcomes remained robust. Complementary multivariable MR analyses showed that none of the potential mediators had statistically significant effects on the GERD-chronic respiratory disease pathway.

In general, caution is required when interpreting the results of MR analysis due to the presence of pleiotropy. The third assumption in MR analysis is the exclusion restriction assumption, meaning that instrumental variables only affect the outcome through the exposure [51]. However, pleiotropy can occur, causing instrumental variables to affect both the outcome variable and other variables through different mechanisms, leading to biased results in MR analysis. Therefore, excluding pleiotropy is a critical prerequisite for MR analysis. While it is challenging to entirely eliminate pleiotropy in practical studies, various sensitivity analysis methods can be used to mitigate its effects and enhance the reliability of the results. As mentioned above, MR-Egger regression and MR-PRESSO method can be applied to evaluate whether the instrumental variable selection results in any potential bias that could substantially impact the results [36, 37]. In our present study, there is a little evidence of pleiotropy for asthma, IPF, and COPD, with P-values ranging from > 0.01 to < 0.05 using the Egger intercept analyses. Meanwhile, MR-PRESSO tests identified two outliers for asthma and one outlier for COPD, but no significant statistical evidence of distortion was found through the distortion tests. Overall, we can conclude from these analyses that horizontal pleiotropy has little or no interference with our causal estimates. It is essential to note that while these methods can alleviate the effects of pleiotropy, they cannot entirely eliminate the possibility of pleiotropic effects. Therefore, the results should be interpreted with caution.

Possible potential mechanisms that have been advanced to explain the causality between GERD and the chronic respiratory diseases we mentioned above. The reflux theory and the reflex15 theory as two main mechanisms have been suggested in the association between GERD and asthma. On the one hand, the reflux theory suggests that micro-aspiration of gastric contents from the proximal esophagus can cause mucosal reaction in the larynx and lungs, leading to respiratory symptoms [52]. On the other hand, the reflex theory describes an indirect mechanism in which distal oesophageal reflux leads to bronchoconstriction secondary to a vagal reflex [53]. Although the reflex theory requires further validation, it is not surprising considering that the esophagus and trachea share the same embryonic origin and innervation by the vagus nerve [54]. The mechanisms underlying the relationship between GERD and COPD and IPF were similar to those in asthma. Furthermore, an increasing number of supporting evidence were detected through the measurement of pepsin and bile salts in broncho alveolar lavage fluid (BALF). Pauwels et al. demonstrated that eight of 29 patients (28%) with asthma were found the presence of bile acids in sputum [55]. Similarly, Savarino E et al. reported higher bile acids and pepsin (*P* < 0.03) in BALF (62% and 67%, respectively) and saliva (61% and 68%, respectively) of IPF patients than that of non-IPF patients (25% and 25% in BALF, and 33% and 36%, respectively, in saliva) and controls (0% and 0% in BALF and saliva, respectively) [47]. Gastric fluid has been shown to induce the expression of matrix metalloproteinase-2 (MMP-2) and MMP-9, as well as activation of the nuclear factor-КB (NF-КB) signaling pathway in macrophages, which can subsequently induce pulmonary fibroblast differentiation [56]. Previous reports have suggested the potentially crucial role of IL-6 produced by macrophages in fibrotic progression and lung disease [57]. Elevated IL-6 expression has been observed in pulmonary fibroblast cells, suggesting that IL-6 may play a potential autocrine or paracrine role in gastric fluid-induced inflammation in the pulmonary microenvironment [58]. In the pathogenesis of asthma, vitamin D is believed to affect lung development, regulate immune responses, and remodel airway smooth muscle [59]. 1,25(OH)_2_D_3_, the active form of vitamin D, has been shown to down-regulate the expression of MMP-9 and improve airway remodeling [60]. In contrast, we did not find a causal association between genetically predicted GERD and vitamin D deficiency, suggesting that GERD may affect smooth muscle cells through remodeling the expression of MMP-2 instead of MMP-9 [57]. Further investigations are needed to confirm these hypotheses.

There are several strengths in our study. First, we conducted the first MR study to assess the causality between GERD and chronic respiratory diseases as well as their risk factors. Participants were grouped according to their randomly allocated genotype, and this procedure mimicked a randomized controlled trial, adding rigor to our findings. In addition, the MR design helps to mitigate issues such as reverse causation and potential confounding factors that are commonly present in conventional observational studies. Secondly, we obtained robustly associated instrumental variables (IVs) from studies with large samples, ensuring that our MR analyses had sufficient statistical power and could provide comparatively accurate estimates of causality.

Several shortcomings in our study cannot be neglected. Firstly, the choice of SNPs and related consortiums were of European origin, which raises concerns about the generalizability of our findings to other populations and regions. Furthermore, the replication group from the FinnGen study did not observe a positive association between GWRD and IPF, which suggests that the current causal results may not be generalizable to them. Additionally, we observed heterogeneity between asthma and COPD in our study. As mentioned previously, the GWASs from which we obtained the summary data of asthma and COPD accepted a wide range of case definitions, which could contribute to the observed heterogeneity. Additionally, we found some sample overlap (UK Biobank mainly) in the GWASs of GERD. Apart from that, comprehensive time-to-event analysis were not conducted to establish the temporal association between GERD and chronic respiratory diseases. Unfortunately, there are no established guidelines for dealing with time-to-event data within an MR context. One analysis using the second stage regression method discussed the relationship between BMI and breast cancer survival, in which survival was considered as the time-to-event outcome [61]. The second stage regression is substituted with either a Cox proportional hazard, or additive hazard regression, which required the outcome to make the Cox model valid [61]. However, the study from which we collected the summary statistics did not provide the necessary data for such analysis. We look forward to future research that will further investigate time-to-event traits and provide more guidance for conducting MR analyses in this context. Other than that, chronic pulmonary diseases have so complex clinical manifestations that its diagnosis should rely on not only used standard ICD codes, but also other examinations or drugs. The diagnosis criteria of our study were cited from corresponding GWAS, which might be inadequate to diagnosis. Future large scale GWAS with more specific inclusion criteria are wanted. Instrumental variables for 12 common risk factors including smoking status (previous and current), exposure to tobacco smoke at home and outside home, obesity class 1–3, alcohol consumption, Type 2 diabetes, Vitamin D deficiency and lung function (FEV1/FVC) were selected from the open MR-base platform ,which all passed the genome-wide significance threshold of p < 5 × 10^− 8^ and the LD analysis with a threshold of R^2^ < 0.001 [21]. Nevertheless, IVs that were not included in the platform need to be explored in future studies.

**In conclusion**, our study provides novel evidence suggesting that individuals with a genetic predisposition to GERD were associated with an increased risk of asthma, IPF, COPD, and chronic bronchitis, while no significant association was observed between GERD and bronchiectasis. The MR results indicated that the micro-aspiration of GERD might play a role in the development of chronic respiratory diseases. Additionally, further large-scale studies are needed to confirm our findings and explore the epidemiological and mechanistic interactions between GERD and chronic respiratory diseases.

## Electronic supplementary material

Below is the link to the electronic supplementary material.

Supplementary Material 1

Supplementary Material 2

## Data Availability

The datasets used and analyzed during the current study available from the corresponding author on reasonable request. R code for the methods proposed in this paper can be found in the package mr.raps that is publicly available at https://github.com/chengbrick/mendelian-randomization-between-GERD-AND-Chronic-respiratory-diseases.git.

## Abbreviations

GERD: Gastroesophageal reflux disease
SNP: Single-nucleotide polymorphism
IPF: Idiopathic pulmonary fibrosis
COPD: Chronic obstructive disease
OR: Odds ratio
CI: Confidence interval
GWASs: Genome-wide association studies
IVW: Inverse variance-weighted
MR: Mendelian randomization
IVs: Instrumental variables
LD: Linkage disequilibrium
