# Supplementary material for: Assessing the genetic relationship between gastroesophageal reflux disease and chronic respiratory diseases: a mendelian randomization study
Source: BMC Pulm Med. 2023 Jul 4;23:243. doi: 10.1186/s12890-023-02502-8 (PMC10318641; doi:10.1186/s12890-023-02502-8)

Figure S1. Leave-one-out analysis for GERD on Asthma.

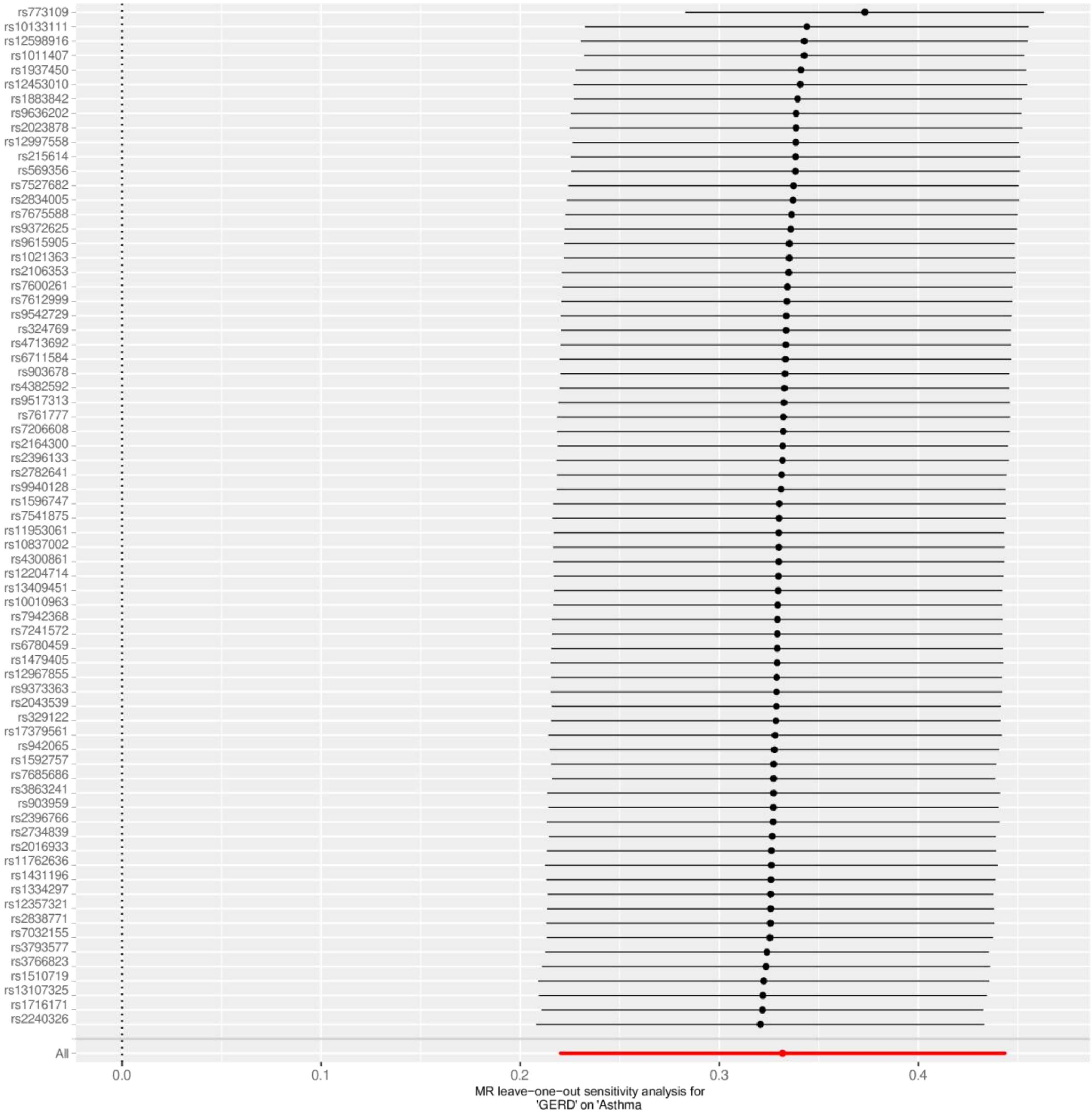

Figure S2. Leave-one-out analysis for GERD on Idiopathic pulmonary fibrosis.

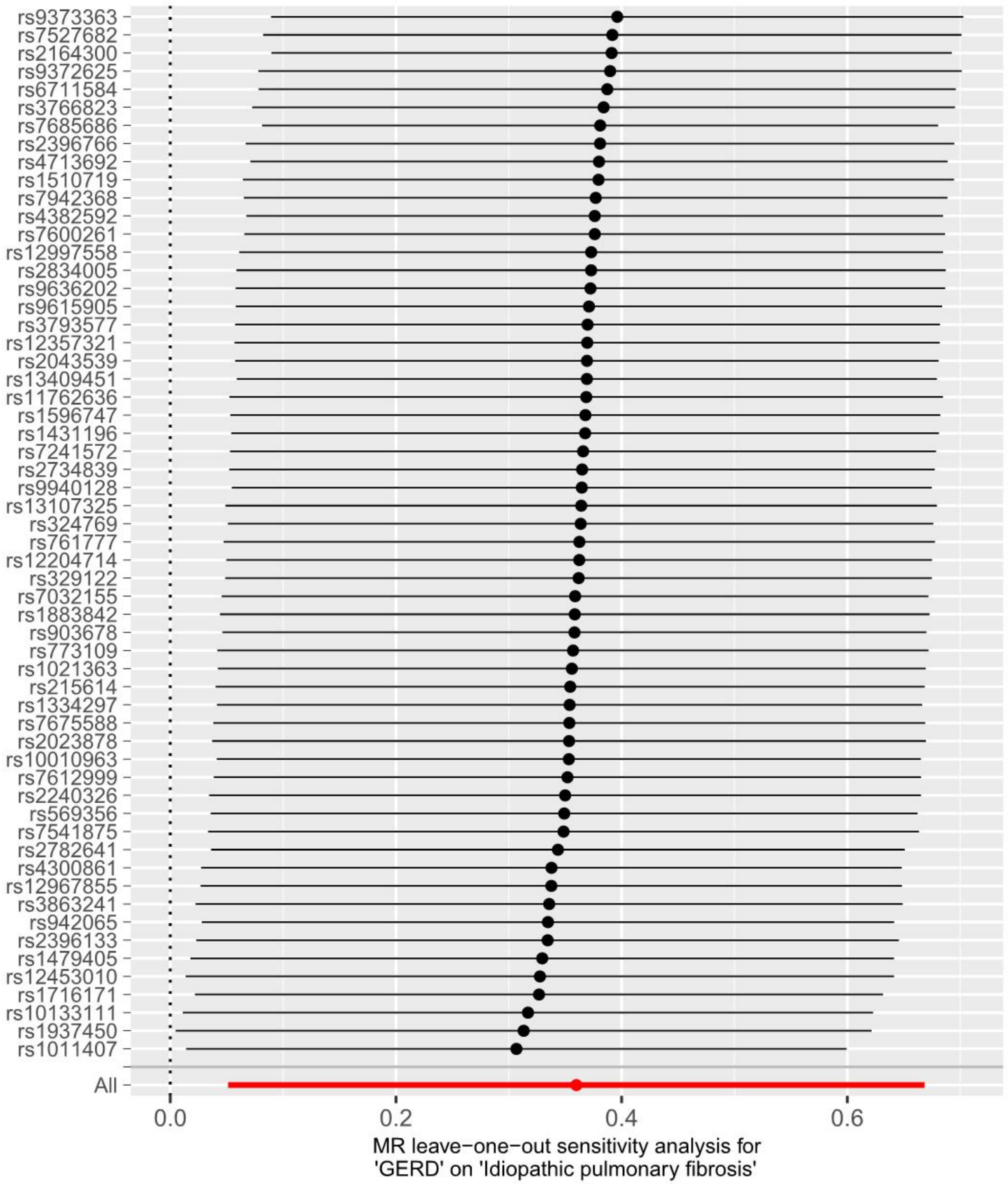

Figure S3. Leave-one-out analysis for GERD on Chronic obstructive pulmonary disease.

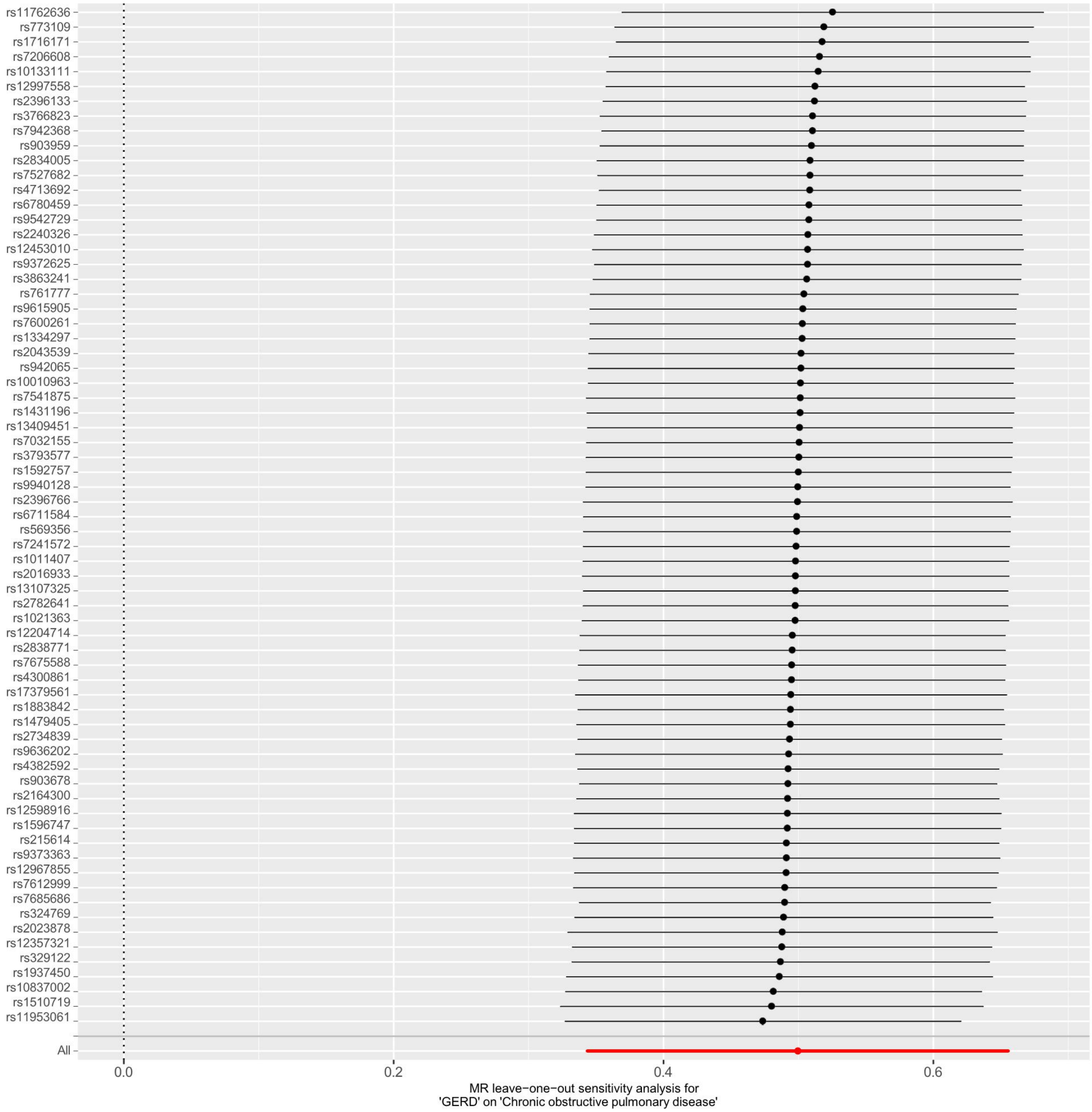

Figure S4. Leave-one-out analysis for GERD on Chronic bronchitis.

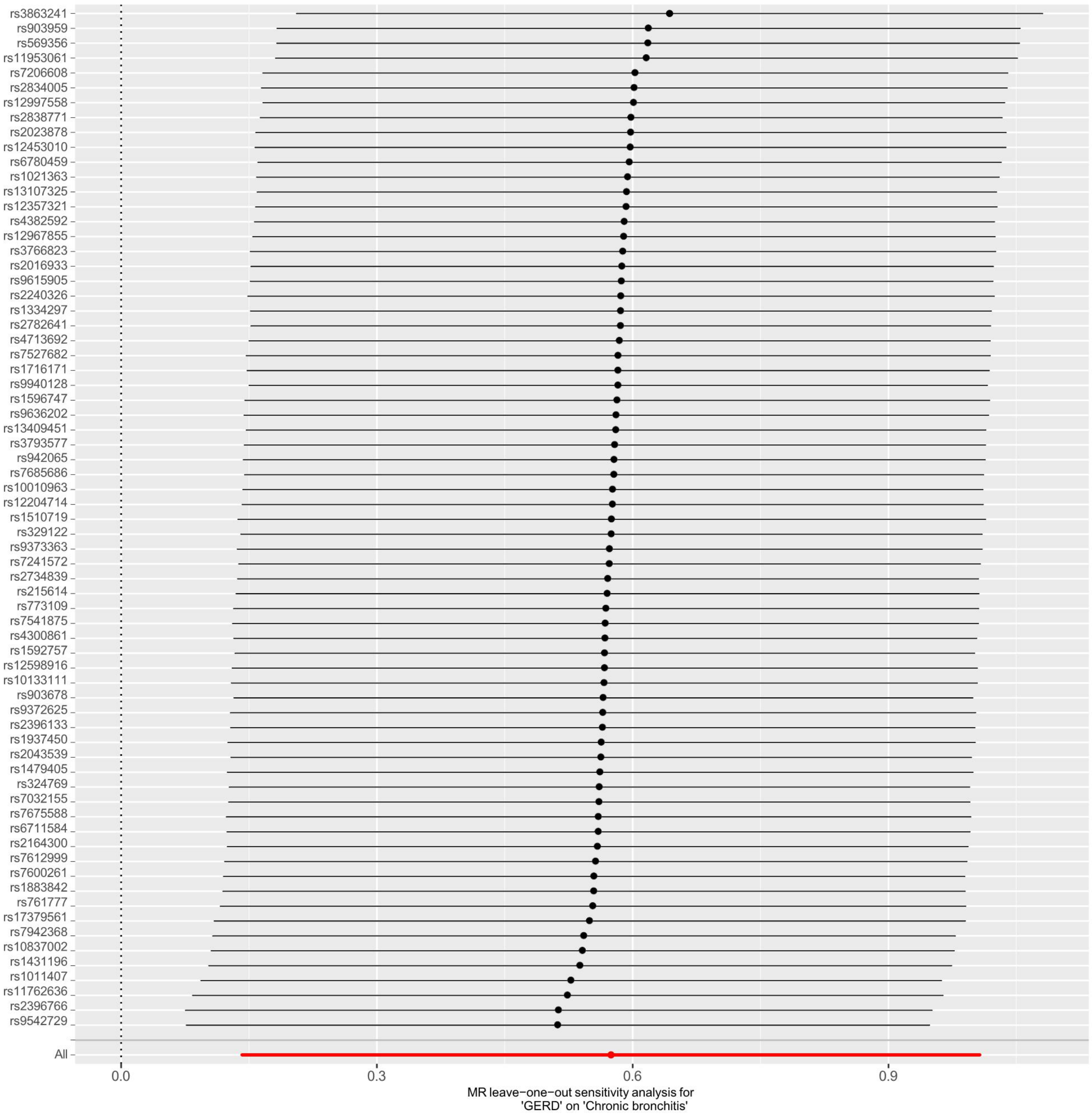

Figure S5. Leave-one-out analysis for GERD on Bronchiectasis.

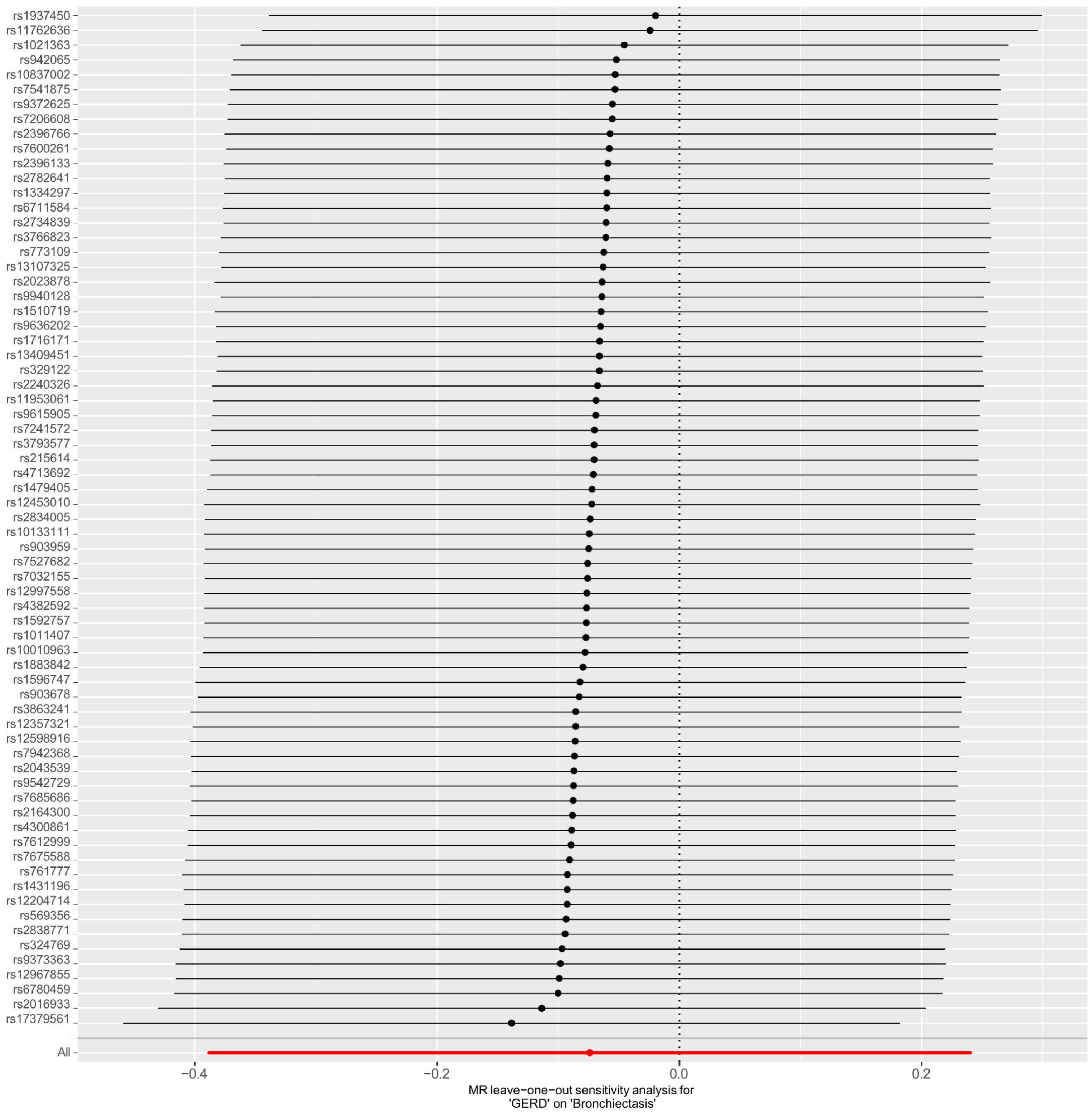

Supplement: Supplementary file 1 — Supplementary Material 1 [file 12890_2023_2502_MOESM1_ESM.pdf]
