# Supplementary material for: Assessing the genetic relationship between gastroesophageal reflux disease and chronic respiratory diseases: a mendelian randomization study
Source: BMC Pulm Med. 2023 Jul 4;23:243. doi: 10.1186/s12890-023-02502-8 (PMC10318641; doi:10.1186/s12890-023-02502-8)
Supplement: Supplementary file 2 — Supplementary Material 2 [file 12890_2023_2502_MOESM2_ESM.pdf]

Supplementary Data

Table S1. Information of 88 risk loci associate with gastroesophageal reflux disease.

SNP	EA	NEA	EAF	Beta	SE	P-value	Chr
rs10010963	C	T	0.383567	0.0166	0.0057	4.92E-08	4
rs1011407	A	G	0.878372	0.0327	0.0085	1.09E-08	2
rs10133111	G	A	0.837004	-0.0358	0.0075	1.35E-10	14
rs1021363	A	G	0.358008	0.0255	0.0058	5.1E-10	10
rs10242223	A	G	0.330202	0.0347	0.0059	3.79E-08	7
rs10789931	C	T	0.882356	-0.0426	0.0087	2.09E-08	11
rs10837002	C	G	0.64878	-0.0256	0.0058	4.03E-08	11
rs11645288	G	A	0.808675	-0.0282	0.0071	2.78E-08	16
rs11762636	C	A	0.819718	0.0413	0.0076	1.88E-16	7
rs11953061	C	T	0.661092	-0.0217	0.0062	0.000000031	5
rs12204714	C	T	0.367777	0.0196	0.0058	7.92E-09	6
rs12357321	G	A	0.688913	-0.0219	0.0061	1.33E-09	10
rs12453010	C	T	0.605197	-0.0385	0.0057	1.75E-09	17
rs12598916	C	G	0.725202	0.0322	0.0063	6.87E-10	16
rs12967855	A	G	0.329565	0.0251	0.006	1.09E-12	18
rs12997558	G	A	0.641249	-0.0215	0.0058	3.04E-08	2
rs13107325	C	T	0.925555	-0.0546	0.0106	2.2E-14	4
rs1334297	G	A	0.265751	0.0205	0.0063	1.14E-12	13
rs13409451	A	G	0.607597	0.0144	0.0057	1.93E-08	2
rs1431196	A	G	0.571568	-0.0236	0.0056	2.65E-11	18
rs1479405	C	T	0.6783	-0.0317	0.006	9.85E-10	12
rs1510719	T	C	0.616561	0.0338	0.0057	3.84E-15	4
rs1592757	G	C	0.644228	-0.0143	0.0058	6E-10	5
rs1596747	A	G	0.505864	-0.0274	0.0056	1E-10	2
rs1716171	C	T	0.209976	-0.0265	0.0068	7.82E-11	12
rs17379561	A	T	0.855609	-0.0451	0.0079	1.08E-14	1
rs17701934	T	C	0.562896	0.0256	0.0056	0.000000046	14
rs1883842	T	G	0.720745	-0.0283	0.0062	9.27E-09	20
rs1937450	T	G	0.462261	-0.0323	0.0056	7.07E-11	1
rs1942262	G	A	0.708574	-0.0272	0.0061	2.6E-09	18
rs2016933	C	G	0.269947	0.0263	0.0063	1.04E-08	3
rs2023878	C	T	0.807623	0.0418	0.0071	3.04E-09	19
rs2043539	G	A	0.58134	-0.0177	0.0056	2.24E-08	7
rs205262	A	G	0.732783	-0.0305	0.0063	1.38E-10	6
rs2106353	G	T	0.768549	-0.038	0.0067	1.37E-10	7
rs2145318	T	A	0.513358	-0.0311	0.0059	2.03E-13	6
rs215614	G	A	0.370275	0.0261	0.0058	4.08E-11	7
rs2164300	C	T	0.476721	0.0179	0.0056	4.13E-08	4
rs2183588	A	G	0.350555	-0.0211	0.0058	1.22E-08	21
rs2240326	G	A	0.526225	0.0306	0.0056	1.13E-22	3
rs2358016	C	G	0.501887	-0.0222	0.0056	4.17E-09	2
rs2396133	A	G	0.524671	-0.0266	0.0056	1.11E-09	7
rs2396766	G	A	0.52692	-0.0307	0.0056	2.33E-11	7
rs2734839	C	T	0.393307	0.0185	0.0057	8.79E-09	11
rs2782641	G	A	0.387331	-0.0145	0.0057	4.33E-08	1
rs2815749	A	G	0.199026	-0.0285	0.007	1.07E-10	1
rs2834005	T	C	0.685	-0.0329	0.006	9.42E-09	21
rs2838771	G	C	0.353279	0.023	0.0058	2.91E-08	21
rs324769	C	T	0.550821	0.0173	0.0056	3.05E-08	12
rs329122	G	A	0.580369	0.0196	0.0056	3.05E-09	5
rs3766823	G	A	0.828533	-0.0347	0.0074	7.09E-10	1
rs3793577	A	G	0.461721	-0.0197	0.0056	2.49E-08	9
rs3863241	C	T	0.47304	-0.0304	0.0056	1.49E-11	8
rs4300861	C	T	0.61792	-0.0237	0.0057	5.43E-10	2
rs4382592	T	G	0.300476	0.0172	0.0061	8.2E-09	9
rs4713692	C	T	0.632192	0.0198	0.0058	3.07E-08	6

rs4851239	C	T	0.620419	0.0345	0.0057	3.24E-11	2
rs569356	A	G	0.859165	0.0338	0.008	4.07E-08	1
rs6441814	G	A	0.529368	0.0264	0.0059	3.86E-09	3
rs6711584	G	A	0.547981	-0.0238	0.0056	2.66E-11	2
rs6780459	A	T	0.253378	-0.0303	0.0064	3.14E-08	3
rs6939294	C	T	0.771219	0.0252	0.0066	2.71E-10	6
rs7032155	C	A	0.40815	-0.02	0.0057	1.63E-08	9
rs7206608	C	G	0.677073	-0.029	0.006	1.46E-08	16
rs7241572	G	A	0.790899	-0.0249	0.007	9.49E-10	18
rs7527682	A	G	0.46275	0.0267	0.0056	3.13E-08	1
rs7541875	A	G	0.573931	-0.0301	0.0056	1.61E-08	1
rs7600261	C	T	0.693609	-0.0211	0.0061	9.47E-11	2
rs7612999	G	A	0.754662	-0.0261	0.0065	0.000000049	3
rs761777	A	G	0.745966	-0.0325	0.0068	4.71E-10	10
rs7675588	C	A	0.205365	0.0364	0.0069	0.000000018	4
rs7685686	A	G	0.577647	0.0117	0.0056	1.14E-08	4
rs773109	G	A	0.664731	0.0305	0.0059	8.71E-14	12
rs7942368	C	T	0.785341	0.0277	0.0069	9.54E-09	11
rs861575	T	C	0.575854	-0.0242	0.0057	1.63E-08	1
rs903678	G	A	0.660588	-0.0128	0.0059	4.89E-08	1
rs903959	T	A	0.600738	-0.0248	0.0057	2.99E-09	8
rs920559	C	G	0.849012	-0.0356	0.0078	2.06E-08	4
rs9372625	G	A	0.616958	0.0303	0.0058	2.62E-14	6
rs9373363	A	G	0.746369	0.0294	0.0065	4.13E-09	6
rs942065	G	A	0.365955	-0.0219	0.0058	8.45E-10	14
rs9517313	G	C	0.616783	-0.0241	0.0057	2.05E-11	13
rs9540720	A	G	0.52114	-0.0265	0.0056	3.01E-08	13
rs9542729	C	G	0.79756	0.0275	0.007	1.41E-09	13
rs957345	C	G	0.459765	-0.026	0.0056	1.72E-11	14
rs9615905	C	T	0.541807	-0.0231	0.0056	1.21E-08	22
rs9636202	G	A	0.73337	0.0324	0.0063	1.51E-10	19
rs9940128	G	A	0.578245	-0.0076	0.0056	8.06E-12	16

SNP: single-nucleotide polymorphism; EA: effect allele; NEA: other allele; EAF: effect allele frequency; SE: standard error; Chr: chromosome.

Table S2. Details of studies of some risk factors for chronic chronic respiratory diseases.

Trait	ID	First author	Consortium	Study participants	Sex	Population	Year	PubmedID
**Smoking status: Previous**	ukb-a-224	Neale	Neale Lab	336024	Males and Females	European	2017	NA
**Smoking status: Current**	ukb-a-225	Neale	Neale Lab	336024	Males and Females	European	2017	NA
**Smoking initiation**	ieu-b-4877	Liu M	GSCAN	607291	Males and Females	European	2019	30643251
**Exposure to tobacco smoke at home**	ukb-a-19	Neale	Neale Lab	305723	Males and Females	European	2017	NA
**Exposure to tobacco smoke outside home**	ukb-a-20	Neale	Neale Lab	286550	Males and Females	European	2017	NA
**Obesity class 1(BMI: 30-34.9 kg/m^2)**	ieu-a-90	Berndt SI	GIANT	98697	Males and Females	European	2013	23563607
**Obesity class 2(BMI: 35-39.9 kg/m^2)**	ieu-a-91	Berndt SI	GIANT	72546	Males and Females	European	2013	23563607
**Obesity class 3(BMI: ≥ 40 kg/ m^2)**	ieu-a-92	Berndt SI	GIANT	50364	Males and Females	European	2013	23563607
**Alcohol consumption**	ieu-a-1283	Clarke	UK Biobank	112117	Males and Females	European	2017	28937693
**Type 2 diabetes**	ebi-a-GCST006867	Xue A	NA	61892	Males and Females	European	2018	30054458
**Vitamin D deficiency**	finn-b-E4_VIT_D_DEF	NA	FinnGen	209789	Males and Females	European	2021	NA
**Lung function (FEV1/FVC)**	ebi-a-GCST007431	Shrine N	NA	321047	Males and Females	European	2019	30804560

Table S3. F-statistics and power estimations for chronic chronic respiratory diseases.

Trait	Number of cases	Number of controls	Samplesize	Sex	Power	F-statistics
**Asthma**	56167	352255	408422	Males and Females	1	1398.09
**Idiopathic pulmonary fibrosis**	4125	20464	24589	Males and Females	1	1387.73
**Chronic obstructive pulmonary disease**	16410	283589	299999	Males and Females	1	1373.25
**Chronic bronchitis**	1035	283589	284624	Males and Females	1	1373.25
**Bronchiectasis**	1967	283589	285556	Males and Females	0.3	1374.25

Table S4. Results of all Mendelian Randomization analyses estimates for associations between GERD and risk of chronic respiratory diseases.

outcome	exposure	method	nsnp	Stand-Error	OR	95% CI		P-value
**Asthma**	GERD	MR Egger	71	0.20	0.91	0.62	1.33	0.628
**Asthma**	GERD	Weighted median	71	0.05	1.36	1.23	1.51	<0.001
**Asthma**	GERD	Inverse variance weighted	71	0.06	1.39	1.25	1.56	<0.001
**Asthma**	GERD	Simple mode	71	0.15	1.64	1.23	2.20	0.001
**Asthma**	GERD	Weighted mode	71	0.11	1.20	0.97	1.49	0.097
**Idiopathic pulmonary fibrosis**	GERD	MR Egger	58	0.53	4.40	1.57	12.34	0.007
**Idiopathic pulmonary fibrosis**	GERD	Weighted median	58	0.21	1.27	0.84	1.93	0.255
**Idiopathic pulmonary fibrosis**	GERD	Inverse variance weighted	58	0.16	1.43	1.05	1.95	0.022
**Idiopathic pulmonary fibrosis**	GERD	Simple mode	58	0.45	1.09	0.45	2.66	0.843
**Idiopathic pulmonary fibrosis**	GERD	Weighted mode	58	0.43	1.20	0.52	2.78	0.666
**Chronic obstructive pulmonary disease**	GERD	MR Egger	69	0.28	0.94	0.55	1.61	0.813
**Chronic obstructive pulmonary disease**	GERD	Weighted median	69	0.09	1.44	1.20	1.73	<0.001
**Chronic obstructive pulmonary disease**	GERD	Inverse variance weighted	69	0.08	1.65	1.41	1.93	<0.001
**Chronic obstructive pulmonary disease**	GERD	Simple mode	69	0.23	1.33	0.85	2.09	0.210
**Chronic obstructive pulmonary disease**	GERD	Weighted mode	69	0.21	1.28	0.84	1.94	0.256
**Chronic bronchitis**	GERD	MR Egger	69	0.78	2.94	0.63	13.65	0.172
**Chronic bronchitis**	GERD	Weighted median	69	0.33	1.84	0.97	3.50	0.062
**Chronic bronchitis**	GERD	Inverse variance weighted	69	0.22	1.78	1.15	2.74	0.009
**Chronic bronchitis**	GERD	Simple mode	69	0.67	1.60	0.43	5.95	0.484
**Chronic bronchitis**	GERD	Weighted mode	69	0.62	1.82	0.54	6.09	0.337
**Bronchiectasis**	GERD	MR Egger	69	0.57	0.86	0.28	2.61	0.785
**Bronchiectasis**	GERD	Weighted median	69	0.23	0.85	0.54	1.34	0.484
**Bronchiectasis**	GERD	Inverse variance weighted	69	0.16	0.93	0.68	1.27	0.645
**Bronchiectasis**	GERD	Simple mode	69	0.50	0.75	0.28	2.00	0.568
**Bronchiectasis**	GERD	Weighted mode	69	0.46	0.71	0.29	1.74	0.455

OR: Odds ratio; CI: Confidence interval.

Table S5. Results of Mendelian Randomization analyses for associations between genetically predicted potential risk factors and chronic respiratory diseases.

Associations between genetically predicted risk factors and Asthma.

outcome	exposure	method	nsnp	OR	95% CI		P-value
**Asthma**	Type 2 diabetes	MR Egger	115	0.99	0.91	1.08	0.786
**Asthma**	Type 2 diabetes	Weighted median	115	0.99	0.95	1.02	0.426
**Asthma**	Type 2 diabetes	Inverse variance weighted	115	1.02	0.98	1.06	0.316
**Asthma**	Type 2 diabetes	Simple mode	115	1.02	0.94	1.10	0.700
**Asthma**	Type 2 diabetes	Weighted mode	115	1.00	0.96	1.04	0.941
**Asthma**	Alcohol consumption	MR Egger	4	0.84	0.13	5.57	0.871
**Asthma**	Alcohol consumption	Weighted median	4	1.05	0.74	1.50	0.789
**Asthma**	Alcohol consumption	Inverse variance weighted	4	1.04	0.76	1.41	0.808
**Asthma**	Alcohol consumption	Simple mode	4	1.12	0.72	1.75	0.652
**Asthma**	Alcohol consumption	Weighted mode	4	1.08	0.73	1.61	0.727
**Asthma**	Obesity class 1(BMI: 30-34.9 kg/m^2)	MR Egger	17	1.09	0.99	1.19	0.086
**Asthma**	Obesity class 1(BMI: 30-34.9 kg/m^2)	Weighted median	17	1.05	1.01	1.10	0.018
**Asthma**	Obesity class 1(BMI: 30-34.9 kg/m2)	Inverse variance weighted	17	1.06	1.03	1.09	<0.001
**Asthma**	Obesity class 1(BMI: 30-34.9 kg/m2)	Simple mode	17	1.07	1.00	1.13	0.056
**Asthma**	Obesity class 1(BMI: 30-34.9 kg/m2)	Weighted mode	17	1.06	1.01	1.12	0.043
**Asthma**	Obesity class 2(BMI: 35-39.9 kg/m2)	MR Egger	11	1.00	0.93	1.08	0.927
**Asthma**	Obesity class 2(BMI: 35-39.9 kg/m2)	Weighted median	11	1.04	1.00	1.07	0.029
**Asthma**	Obesity class 2(BMI: 35-39.9 kg/m2)	Inverse variance weighted	11	1.05	1.02	1.08	<0.001
**Asthma**	Obesity class 2(BMI: 35-39.9 kg/m2)	Simple mode	11	1.04	0.99	1.10	0.155
**Asthma**	Obesity class 2(BMI: 35-39.9 kg/m2)	Weighted mode	11	1.04	1.00	1.08	0.088
**Asthma**	Obesity class 3(BMI: ≥ 40 kg/ m2)	Inverse variance weighted	2	1.02	0.98	1.05	0.329
**Asthma**	Smoking initiation	MR Egger	84	0.88	0.53	1.44	0.602
**Asthma**	Smoking initiation	Weighted median	84	1.17	1.06	1.29	0.002
**Asthma**	Smoking initiation	Inverse variance weighted	84	1.16	1.05	1.28	0.003
**Asthma**	Smoking initiation	Simple mode	84	1.25	0.99	1.59	0.062
**Asthma**	Smoking initiation	Weighted mode	84	1.22	0.98	1.52	0.075
**Asthma**	Exposure to tobacco smoke at home	Wald ratio	1	1.46	0.14	15.09	0.753
**Asthma**	Exposure to tobacco smoke outside home	Inverse variance weighted	2	1.83	0.32	10.39	0.495
**Asthma**	Smoking status: Previous	MR Egger	18	0.65	0.01	31.63	0.831
**Asthma**	Smoking status: Previous	Weighted median	18	1.50	0.82	2.74	0.193
**Asthma**	Smoking status: Previous	Inverse variance weighted	18	1.01	0.52	1.97	0.978
**Asthma**	Smoking status: Previous	Simple mode	18	1.72	0.62	4.84	0.314
**Asthma**	Smoking status: Previous	Weighted mode	18	1.67	0.59	4.72	0.350
**Asthma**	Smoking status: Current	MR Egger	15	50.28	0.26	9591.88	0.167
**Asthma**	Smoking status: Current	Weighted median	15	0.93	0.33	2.60	0.887
**Asthma**	Smoking status: Current	Inverse variance weighted	15	0.78	0.22	2.74	0.693
**Asthma**	Smoking status: Current	Simple mode	15	0.74	0.13	4.14	0.740
**Asthma**	Smoking status: Current	Weighted mode	15	0.81	0.13	4.86	0.819

Associations between genetically predicted risk factors and Idiopathic pulmonary fibrosis.

outcome	exposure	method	nsnp	OR	95% CI		P-value
**Idiopathic**	Type 2 diabetes	MR Egger	107	1.16	0.95	1.42	0.146

**pulmonary fibrosis**							
**Idiopathic pulmonary fibrosis**	Type 2 diabetes	Weighted median	107	1.10	0.95	1.27	0.190
**Idiopathic pulmonary fibrosis**	Type 2 diabetes	Inverse variance weighted	107	0.99	0.90	1.08	0.814
**Idiopathic pulmonary fibrosis**	Type 2 diabetes	Simple mode	107	0.93	0.70	1.23	0.617
**Idiopathic pulmonary fibrosis**	Type 2 diabetes	Weighted mode	107	1.09	0.95	1.25	0.208
**Idiopathic pulmonary fibrosis**	Alcohol consumption	MR Egger	240	12.31	0.00	44263.58	0.655
**Idiopathic pulmonary fibrosis**	Alcohol consumption	Weighted median	240	0.58	0.13	2.51	0.463
**Idiopathic pulmonary fibrosis**	Alcohol consumption	Inverse variance weighted	240	0.55	0.15	2.04	0.368
**Idiopathic pulmonary fibrosis**	Alcohol consumption	Simple mode	240	0.43	0.06	2.93	0.477
**Idiopathic pulmonary fibrosis**	Alcohol consumption	Weighted mode	240	0.67	0.12	3.65	0.685
**Idiopathic pulmonary fibrosis**	Obesity class 1(BMI: 30-34.9 kg/m^2)	MR Egger	3	1.25	0.78	2.00	0.382
**Idiopathic pulmonary fibrosis**	Obesity class 1(BMI: 30-34.9 kg/m^2)	Weighted median	3	1.04	0.84	1.29	0.720
**Idiopathic pulmonary fibrosis**	Obesity class 1(BMI: 30-34.9 kg/m^2)	Inverse variance weighted	3	1.05	0.88	1.24	0.605
**Idiopathic pulmonary fibrosis**	Obesity class 1(BMI: 30-34.9 kg/m^2)	Simple mode	3	1.16	0.81	1.67	0.425
**Idiopathic pulmonary fibrosis**	Obesity class 1(BMI: 30-34.9 kg/m^2)	Weighted mode	3	1.06	0.81	1.38	0.688
**Idiopathic pulmonary fibrosis**	Obesity class 2(BMI: 35-39.9 kg/m^2)	MR Egger	14	1.03	0.65	1.64	0.893
**Idiopathic pulmonary fibrosis**	Obesity class 2(BMI: 35-39.9 kg/m^2)	Weighted median	14	0.96	0.83	1.12	0.621
**Idiopathic pulmonary fibrosis**	Obesity class 2(BMI: 35-39.9 kg/m^2)	Inverse variance weighted	14	1.07	0.92	1.23	0.374
**Idiopathic pulmonary fibrosis**	Obesity class 2(BMI: 35-39.9 kg/m^2)	Simple mode	14	0.96	0.75	1.23	0.768
**Idiopathic pulmonary fibrosis**	Obesity class 2(BMI: 35-39.9 kg/m^2)	Weighted mode	14	0.95	0.80	1.13	0.598
**Idiopathic pulmonary fibrosis**	Obesity class 3(BMI:≥ 40 kg/ m^2)	Inverse variance weighted	11	0.99	0.87	1.13	0.900
**Idiopathic pulmonary fibrosis**	Smoking initiation	MR Egger	11	0.36	0.05	2.86	0.340
**Idiopathic pulmonary fibrosis**	Smoking initiation	Weighted median	11	0.87	0.57	1.32	0.504
**Idiopathic pulmonary fibrosis**	Smoking initiation	Inverse variance weighted	11	1.09	0.73	1.63	0.676
**Idiopathic**	Smoking initiation	Simple mode	11	0.60	0.24	1.50	0.276

**pulmonary fibrosis**							
**Idiopathic pulmonary fibrosis**	Smoking initiation	Weighted mode	2	0.66	0.29	1.51	0.324
**Idiopathic pulmonary fibrosis**	Exposure to tobacco smoke at home	Wald ratio	71	26.54	0.00	662036.21	0.526
**Idiopathic pulmonary fibrosis**	Exposure to tobacco smoke outside home	Inverse variance weighted	71	135.71	0.15	124182.66	0.158
**Idiopathic pulmonary fibrosis**	Smoking status: Previous	MR Egger	71	0.02	0.00	8094064.47	0.715
**Idiopathic pulmonary fibrosis**	Smoking status: Previous	Weighted median	71	2.16	0.15	30.62	0.570
**Idiopathic pulmonary fibrosis**	Smoking status: Previous	Inverse variance weighted	71	2.24	0.21	23.65	0.503
**Idiopathic pulmonary fibrosis**	Smoking status: Previous	Simple mode	1	0.48	0.00	82.81	0.785
**Idiopathic pulmonary fibrosis**	Smoking status: Previous	Weighted mode	1	0.55	0.00	72.12	0.811
**Idiopathic pulmonary fibrosis**	Smoking status: Current	MR Egger	17	0.00	0.00	758.96	0.305
**Idiopathic pulmonary fibrosis**	Smoking status: Current	Weighted median	17	0.24	0.00	23.17	0.544
**Idiopathic pulmonary fibrosis**	Smoking status: Current	Inverse variance weighted	17	0.10	0.00	2.25	0.145
**Idiopathic pulmonary fibrosis**	Smoking status: Current	Simple mode	17	3.21	0.00	46127.99	0.815
**Idiopathic pulmonary fibrosis**	Smoking status: Current	Weighted mode	17	6.10	0.00	76124.42	0.713

Associations between genetically predicted risk factors and Chronic obstructive pulmonary disease.

outcome	exposure	method	nsnp	OR	95% CI		P-value
**Chronic obstructive pulmonary disease**	Type 2 diabetes	MR Egger	113	0.91	0.82	1.00	0.056
**Chronic obstructive pulmonary disease**	Type 2 diabetes	Weighted median	113	0.95	0.89	1.02	0.161
**Chronic obstructive pulmonary disease**	Type 2 diabetes	Inverse variance weighted	113	0.99	0.95	1.03	0.515
**Chronic obstructive pulmonary disease**	Type 2 diabetes	Simple mode	113	0.97	0.84	1.12	0.684
**Chronic obstructive pulmonary disease**	Type 2 diabetes	Weighted mode	113	0.96	0.89	1.03	0.278
**Chronic obstructive pulmonary disease**	Alcohol consumption	MR Egger	4	27.01	0.07	10724.85	0.393
**Chronic obstructive pulmonary disease**	Alcohol consumption	Weighted median	4	0.83	0.41	1.70	0.617
**Chronic obstructive pulmonary disease**	Alcohol consumption	Inverse variance weighted	4	0.79	0.29	2.15	0.642
**Chronic obstructive pulmonary disease**	Alcohol consumption	Simple mode	4	0.58	0.19	1.76	0.411
**Chronic obstructive**	Alcohol consumption	Weighted mode	4	0.86	0.38	1.92	0.731

**pulmonary disease**							
**Chronic obstructive pulmonary disease**	Obesity class 1(BMI: 30-34.9 kg/m^2)	MR Egger	16	1.07	0.88	1.30	0.502
**Chronic obstructive pulmonary disease**	Obesity class 1(BMI: 30-34.9 kg/m^2)	Weighted median	16	0.99	0.91	1.09	0.886
**Chronic obstructive pulmonary disease**	Obesity class 1(BMI: 30-34.9 kg/m^2)	Inverse variance weighted	16	1.03	0.96	1.10	0.479
**Chronic obstructive pulmonary disease**	Obesity class 1(BMI: 30-34.9 kg/m^2)	Simple mode	16	0.96	0.83	1.11	0.590
**Chronic obstructive pulmonary disease**	Obesity class 1(BMI: 30-34.9 kg/m^2)	Weighted mode	16	0.99	0.90	1.09	0.836
**Chronic obstructive pulmonary disease**	Obesity class 2(BMI: 35-39.9 kg/m^2)	MR Egger	11	1.06	0.90	1.26	0.487
**Chronic obstructive pulmonary disease**	Obesity class 2(BMI: 35-39.9 kg/m^2)	Weighted median	11	1.02	0.95	1.09	0.675
**Chronic obstructive pulmonary disease**	Obesity class 2(BMI: 35-39.9 kg/m^2)	Inverse variance weighted	11	1.03	0.98	1.09	0.292
**Chronic obstructive pulmonary disease**	Obesity class 2(BMI: 35-39.9 kg/m^2)	Simple mode	11	1.06	0.94	1.20	0.358
**Chronic obstructive pulmonary disease**	Obesity class 2(BMI: 35-39.9 kg/m^2)	Weighted mode	11	1.02	0.94	1.10	0.665
**Chronic obstructive pulmonary disease**	Obesity class 3(BMI:≥ 40 kg/ m^2)	Inverse variance weighted	2	0.98	0.93	1.04	0.593
**Chronic obstructive pulmonary disease**	Smoking initiation	MR Egger	83	2.29	1.07	4.88	0.036
**Chronic obstructive pulmonary disease**	Smoking initiation	Weighted median	83	1.71	1.42	2.05	<0.001
**Chronic obstructive pulmonary disease**	Smoking initiation	Inverse variance weighted	83	1.72	1.48	2.00	<0.001
**Chronic obstructive pulmonary disease**	Smoking initiation	Simple mode	83	1.27	0.72	2.25	0.411
**Chronic obstructive pulmonary disease**	Smoking initiation	Weighted mode	83	1.56	0.95	2.57	0.081
**Chronic obstructive pulmonary disease**	Exposure to tobacco smoke at home	Wald ratio	1	0.10	0.00	3.75	0.213
**Chronic obstructive pulmonary disease**	Exposure to tobacco smoke outside home	Inverse variance weighted	2	5.92	1.76	19.93	0.004
**Chronic obstructive pulmonary disease**	Smoking status: Previous	MR Egger	18	0.02	0.00	2.96	0.142
**Chronic obstructive pulmonary disease**	Smoking status: Previous	Weighted median	18	0.79	0.26	2.41	0.683
**Chronic obstructive pulmonary disease**	Smoking status: Previous	Inverse variance weighted	18	0.47	0.19	1.20	0.115
**Chronic obstructive pulmonary disease**	Smoking status: Previous	Simple mode	18	0.96	0.10	9.33	0.970
**Chronic obstructive pulmonary disease**	Smoking status: Previous	Weighted mode	18	0.88	0.09	8.32	0.912
**Chronic obstructive**	Smoking status: Current	MR Egger	15	1665.23	0.00	2216941	0.451

**pulmonary disease**						56760.41	
**Chronic obstructive pulmonary disease**	Smoking status: Current	Weighted median	15	264.52	26.07	2683.99	<0.001
**Chronic obstructive pulmonary disease**	Smoking status: Current	Inverse variance weighted	15	135.63	5.93	3100.91	0.002
**Chronic obstructive pulmonary disease**	Smoking status: Current	Simple mode	15	459.29	16.91	12475.81	0.003
**Chronic obstructive pulmonary disease**	Smoking status: Current	Weighted mode	15	368.10	9.52	14230.92	0.007

Associations between genetically predicted risk factors and Chronic bronchitis.

outcome	exposure	method	nsnp	OR	95% CI		P-value
**Chronic bronchitis**	Type 2 diabetes	MR Egger	113	1.00	0.73	1.36	0.989
**Chronic bronchitis**	Type 2 diabetes	Weighted median	113	1.03	0.82	1.30	0.772
**Chronic bronchitis**	Type 2 diabetes	Inverse variance weighted	113	0.91	0.80	1.03	0.146
**Chronic bronchitis**	Type 2 diabetes	Simple mode	113	1.14	0.75	1.74	0.539
**Chronic bronchitis**	Type 2 diabetes	Weighted mode	113	1.05	0.81	1.37	0.694
**Chronic bronchitis**	Alcohol consumption	MR Egger	4	0.00	0.00	5993.38	0.482
**Chronic bronchitis**	Alcohol consumption	Weighted median	4	0.34	0.02	5.35	0.441
**Chronic bronchitis**	Alcohol consumption	Inverse variance weighted	4	0.25	0.03	2.29	0.218
**Chronic bronchitis**	Alcohol consumption	Simple mode	4	0.83	0.02	28.94	0.923
**Chronic bronchitis**	Alcohol consumption	Weighted mode	4	0.50	0.03	9.61	0.680
**Chronic bronchitis**	Obesity class 1(BMI: 30-34.9 kg/m^2)	MR Egger	16	0.98	0.52	1.83	0.949
**Chronic bronchitis**	Obesity class 1(BMI: 30-34.9 kg/m^2)	Weighted median	16	0.92	0.67	1.26	0.610
**Chronic bronchitis**	Obesity class 1(BMI: 30-34.9 kg/m^2)	Inverse variance weighted	16	0.89	0.71	1.11	0.286
**Chronic bronchitis**	Obesity class 1(BMI: 30-34.9 kg/m^2)	Simple mode	16	1.04	0.67	1.61	0.856
**Chronic bronchitis**	Obesity class 1(BMI: 30-34.9 kg/m^2)	Weighted mode	16	0.93	0.65	1.33	0.696
**Chronic bronchitis**	Obesity class 2(BMI: 35-39.9 kg/m^2)	MR Egger	11	0.81	0.48	1.37	0.451
**Chronic bronchitis**	Obesity class 2(BMI: 35-39.9 kg/m^2)	Weighted median	11	0.92	0.73	1.17	0.504
**Chronic bronchitis**	Obesity class 2(BMI: 35-39.9 kg/m^2)	Inverse variance weighted	11	0.92	0.77	1.09	0.326
**Chronic bronchitis**	Obesity class 2(BMI: 35-39.9 kg/m^2)	Simple mode	11	1.12	0.77	1.64	0.559
**Chronic bronchitis**	Obesity class 2(BMI: 35-39.9 kg/m^2)	Weighted mode	11	0.93	0.70	1.22	0.602
**Chronic bronchitis**	Obesity class 3(BMI:≥ 40 kg/ m^2)	Inverse variance weighted	2	0.92	0.74	1.13	0.431
**Chronic bronchitis**	Smoking initiation	MR Egger	83	1.14	0.13	10.10	0.904
**Chronic bronchitis**	Smoking initiation	Weighted median	83	1.18	0.65	2.14	0.592
**Chronic bronchitis**	Smoking initiation	Inverse variance weighted	83	1.34	0.87	2.06	0.186
**Chronic bronchitis**	Smoking initiation	Simple mode	83	0.75	0.16	3.40	0.706
**Chronic bronchitis**	Smoking initiation	Weighted mode	83	0.61	0.13	2.74	0.518
**Chronic bronchitis**	Exposure to tobacco smoke at home	Wald ratio	1	0.30	0.00	190832.97	0.861
**Chronic bronchitis**	Exposure to tobacco smoke outside home	Inverse variance weighted	2	2.07	0.02	183.01	0.750
**Chronic bronchitis**	Smoking status: Previous	MR Egger	18	92222.11	0.02	553509754454.87	0.170
**Chronic bronchitis**	Smoking status: Previous	Weighted median	18	2.52	0.06	114.18	0.634
**Chronic bronchitis**	Smoking status: Previous	Inverse variance weighted	18	2.87	0.18	46.91	0.459
**Chronic bronchitis**	Smoking status: Previous	Simple mode	18	1.98	0.00	830.00	0.827
**Chronic bronchitis**	Smoking status: Previous	Weighted mode	18	1.69	0.00	686.30	0.866
**Chronic bronchitis**	Smoking status: Current	MR Egger	15	971461	0.97	9716816	0.072

				396343.18		08882018000000000.00	
**Chronic bronchitis**	Smoking status: Current	Weighted median	15	0.11	0.00	131.99	0.543
**Chronic bronchitis**	Smoking status: Current	Inverse variance weighted	15	30.63	0.18	5097.05	0.190
**Chronic bronchitis**	Smoking status: Current	Simple mode	15	0.04	0.00	3216.19	0.583
**Chronic bronchitis**	Smoking status: Current	Weighted mode	15	0.03	0.00	2033.19	0.551

Table S6. The exact outcomes of Mendelian randomization pleiotropy test from egger regression.

outcome	exposure	Intercept	Standard error	P-value
**Asthma**	GERD	0.0118	0.0052	0.0261
**Idiopathic pulmonary fibrosis**	GERD	-0.0308	0.0138	0.0298
**Chronic obstructive pulmonary disease**	GERD	0.0155	0.0073	0.0360
**Chronic bronchitis**	GERD	-0.0139	0.0207	0.5035
**Bronchiectasis**	GERD	0.0023	0.0150	0.8808

Table S7. MR-PRESSO tests of 5 chronic respiratory diseases.

outcomes	OR	95% CI		P-value	Global Test P-value	Outlier number	Distortion Test P-value
**Asthma**	1.46	1.33	1.59	<0.0001	<0.0005	2	0.239
**Idiopathic pulmonary fibrosis**	1.50	1.14	1.99	0.006	0.178	0	NA
**Chronic obstructive pulmonary disease**	1.62	1.41	1.87	<0.0001	<0.0005	1	0.709
**Chronic bronchitis**	1.83	1.25	2.67	0.003	0.897	0	NA
**Bronchiectasis**	0.96	0.73	1.28	0.803	0.829	0	NA

Table S8. The exact outcomes of Mendelian randomization heterogeneity test.

exposure	method	cochran's Q test	degree of Freedom	p-value
**GERD**	MR Egger	209.5842	69.0000	<0.0001
**GERD**	Inverse variance weighted	225.2766	70.0000	<0.0001
**GERD**	MR Egger	58.9945	56.0000	0.3666
**GERD**	Inverse variance weighted	64.2341	57.0000	0.2380
**GERD**	MR Egger	115.1888	67.0000	0.0002
**GERD**	Inverse variance weighted	123.0657	68.0000	<0.0001
**GERD**	MR Egger	53.0435	67.0000	0.8930
**GERD**	Inverse variance weighted	53.4958	68.0000	0.9009
**GERD**	MR Egger	58.0547	67.0000	0.7739
**GERD**	Inverse variance weighted	58.0774	68.0000	0.7991

Table S9. The associations between individual SNP and risks of five pulmonary diseases.

exposure	Outcome	SNP	Beta	Standard error	P-value
**GERD**	Asthma	rs10010963	0.73489759	0.404216867	0.069052289
**GERD**	Asthma	rs1011407	-0.658819572	0.305043731	0.030791679
**GERD**	Asthma	rs10133111	-0.384184358	0.246280726	0.118772908
**GERD**	Asthma	rs1021363	0.100482745	0.267159608	0.706831378
**GERD**	Asthma	rs10837002	0.465710938	0.266629688	0.08069664
**GERD**	Asthma	rs11762636	0.565312349	0.205877482	0.006035108
**GERD**	Asthma	rs11953061	0.522976959	0.318452995	0.100539813
**GERD**	Asthma	rs12204714	0.576193878	0.345118367	0.095007565
**GERD**	Asthma	rs12357321	0.959424658	0.324173059	0.003080323
**GERD**	Asthma	rs12453010	0.075311169	0.174086494	0.665299747
**GERD**	Asthma	rs12598916	-0.215049068	0.227232609	0.343953295
**GERD**	Asthma	rs12967855	0.563988048	0.277381275	0.042026336
**GERD**	Asthma	rs12997558	-0.31722186	0.316915349	0.31684268
**GERD**	Asthma	rs13107325	0.830635531	0.227261905	0.000257212
**GERD**	Asthma	rs1334297	1.105102439	0.360290244	0.002160337
**GERD**	Asthma	rs13409451	0.8133125	0.464835417	0.080173818
**GERD**	Asthma	rs1431196	0.786538136	0.279875847	0.004949367
**GERD**	Asthma	rs1479405	0.464962145	0.220263407	0.034777739
**GERD**	Asthma	rs1510719	0.693825444	0.198775444	0.00048212
**GERD**	Asthma	rs1592757	1.336160839	0.47596014	0.004995936
**GERD**	Asthma	rs1596747	0.424412409	0.238082482	0.07464694
**GERD**	Asthma	rs1716171	1.246411321	0.302778113	3.85E-05
**GERD**	Asthma	rs17379561	0.489751663	0.20583592	0.017344122
**GERD**	Asthma	rs1883842	-0.159864664	0.257340989	0.534456685
**GERD**	Asthma	rs1937450	-0.036586997	0.204181424	0.857789563
**GERD**	Asthma	rs2016933	0.764475285	0.279468441	0.006229279
**GERD**	Asthma	rs2023878	0.07806244	0.198170096	0.693642801
**GERD**	Asthma	rs2043539	0.775372881	0.37320678	0.037746593
**GERD**	Asthma	rs2106353	0.208136053	0.204322105	0.308361394
**GERD**	Asthma	rs215614	-0.091413793	0.258884291	0.724008406
**GERD**	Asthma	rs2164300	0.314064246	0.366140782	0.39101998
**GERD**	Asthma	rs2240326	0.828666667	0.213348366	0.000102709
**GERD**	Asthma	rs2396133	0.330519549	0.245854135	0.178827948
**GERD**	Asthma	rs2396766	0.538416938	0.213055049	0.011500026
**GERD**	Asthma	rs2734839	1.000345946	0.361159459	0.005608764
**GERD**	Asthma	rs2782641	0.431749655	0.463764138	0.351870025
**GERD**	Asthma	rs2834005	0.102183587	0.213132827	0.631628366
**GERD**	Asthma	rs2838771	0.863678261	0.29668087	0.003601176
**GERD**	Asthma	rs324769	0.095176879	0.379281503	0.801860542
**GERD**	Asthma	rs329122	0.722591837	0.337781633	0.032417229
**GERD**	Asthma	rs3766823	0.838244957	0.249340346	0.00077422
**GERD**	Asthma	rs3793577	1.199045685	0.335312183	0.000349014
**GERD**	Asthma	rs3863241	0.534769737	0.214736513	0.012761625
**GERD**	Asthma	rs4300861	0.484738397	0.2835827	0.087388547

**GERD**	Asthma	rs4382592	0.178649419	0.413618023	0.665800398
**GERD**	Asthma	rs4713692	0.153009091	0.344163131	0.656621107
**GERD**	Asthma	rs569356	-0.149159467	0.277188462	0.590497163
**GERD**	Asthma	rs6711584	0.230660084	0.276373109	0.403944823
**GERD**	Asthma	rs6780459	0.497046205	0.24759835	0.044699665
**GERD**	Asthma	rs7032155	1.033475	0.3335835	0.001947658
**GERD**	Asthma	rs7206608	0.309595172	0.240627586	0.198228353
**GERD**	Asthma	rs7241572	0.615196787	0.326548193	0.059573264
**GERD**	Asthma	rs7527682	0.01131603	0.245127715	0.963179685
**GERD**	Asthma	rs7541875	0.414986711	0.21881794	0.057894873
**GERD**	Asthma	rs7600261	0.064538863	0.335256398	0.847345882
**GERD**	Asthma	rs7612999	0.155854789	0.290624521	0.591768211
**GERD**	Asthma	rs761777	0.308649231	0.229922462	0.179464477
**GERD**	Asthma	rs7675588	0.120677747	0.221842308	0.586455333
**GERD**	Asthma	rs7685686	1.760589744	0.56507265	0.001835167
**GERD**	Asthma	rs773109	-1.725137705	0.226491803	2.60E-14
**GERD**	Asthma	rs7942368	0.551967509	0.291309386	0.058121413
**GERD**	Asthma	rs903678	-0.002695086	0.538645313	0.99600784
**GERD**	Asthma	rs903959	0.658572581	0.268897177	0.014318928
**GERD**	Asthma	rs9372625	0.140241584	0.222253135	0.528040949
**GERD**	Asthma	rs9373363	0.534030612	0.25697517	0.037696383
**GERD**	Asthma	rs942065	0.722484018g	0.310332877	0.019907128
**GERD**	Asthma	rs9517313	0.280424896	0.277958091	0.313034712
**GERD**	Asthma	rs9542729	0.181605818	0.295742545	0.539170874
**GERD**	Asthma	rs9615905	0.067384416	0.283116883	0.811873979
**GERD**	Asthma	rs9636202	-0.010946235	0.228451543	0.961784059
**GERD**	Asthma	rs9940128	0.904089474	0.868476316	0.29787254
**GERD**	Asthma	All - Inverse variance weighted	0.331862132	0.05696878	5.70E-09
**GERD**	Asthma	All - MR Egger	-0.095414208	0.195961457	0.627870292
**GERD**	Idiopathic pulmonary fibrosis	rs10010963	1.21686747	1.682426777	0.469507209
**GERD**	Idiopathic pulmonary fibrosis	rs1011407	4.443425076	1.308508858	0.000684301
**GERD**	Idiopathic pulmonary fibrosis	rs10133111	2.226256983	0.989132876	0.024403544
**GERD**	Idiopathic pulmonary fibrosis	rs1021363	0.619607843	1.19855013	0.605180955
**GERD**	Idiopathic pulmonary fibrosis	rs11762636	0.092009685	0.834441869	0.912199259
**GERD**	Idiopathic pulmonary fibrosis	rs12204714	0.081632653	1.570092236	0.958534856
**GERD**	Idiopathic pulmonary fibrosis	rs12357321	-0.410958904	1.33782426	0.758702927
**GERD**	Idiopathic pulmonary fibrosis	rs12453010	1.233766234	0.785632703	0.116319526
**GERD**	Idiopathic pulmonary fibrosis	rs12967855	1.665338645	1.146271017	0.146270543
**GERD**	Idiopathic pulmonary fibrosis	rs12997558	-0.646511628	1.308793675	0.621323688
**GERD**	Idiopathic pulmonary fibrosis	rs13107325	0.192307692	0.940014828	0.837900733
**GERD**	Idiopathic pulmonary fibrosis	rs1334297	0.975609756	1.506302321	0.517188563
**GERD**	Idiopathic pulmonary fibrosis	rs13409451	-1.506944444	2.107795056	0.474646868
**GERD**	Idiopathic pulmonary fibrosis	rs1431196	-0.101694915	1.156607881	0.929936171
**GERD**	Idiopathic pulmonary fibrosis	rs1479405	1.479495268	0.914264417	0.105611824
**GERD**	Idiopathic pulmonary fibrosis	rs1510719	-0.236686391	0.832516686	0.77617874
**GERD**	Idiopathic pulmonary fibrosis	rs1596747	0.01459854	0.988509725	0.988217085
**GERD**	Idiopathic pulmonary fibrosis	rs1716171	2.690566038	1.252746118	0.031734854

**GERD**	Idiopathic pulmonary fibrosis	rs1883842	0.434628975	1.076241645	0.686330688
**GERD**	Idiopathic pulmonary fibrosis	rs1937450	1.842105263	0.848338568	0.02989891
**GERD**	Idiopathic pulmonary fibrosis	rs2023878	0.559808612	0.83454565	0.502351467
**GERD**	Idiopathic pulmonary fibrosis	rs2043539	-0.666666667	1.560006511	0.669125407
**GERD**	Idiopathic pulmonary fibrosis	rs215614	0.643678161	1.074087136	0.548986665
**GERD**	Idiopathic pulmonary fibrosis	rs2164300	-2.882681564	1.519025488	0.057733279
**GERD**	Idiopathic pulmonary fibrosis	rs2240326	0.709150327	0.888580269	0.42482917
**GERD**	Idiopathic pulmonary fibrosis	rs2396133	1.545112782	1.022201362	0.130647305
**GERD**	Idiopathic pulmonary fibrosis	rs2396766	-0.377850163	0.892551653	0.672049383
**GERD**	Idiopathic pulmonary fibrosis	rs2734839	-0.156756757	1.509637	0.917298399
**GERD**	Idiopathic pulmonary fibrosis	rs2782641	3.082758621	1.918820327	0.108144215
**GERD**	Idiopathic pulmonary fibrosis	rs2834005	-0.188449848	0.973814129	0.846553885
**GERD**	Idiopathic pulmonary fibrosis	rs324769	-0.069364162	1.589984132	0.96520277
**GERD**	Idiopathic pulmonary fibrosis	rs329122	0.183673469	1.403404362	0.895872544
**GERD**	Idiopathic pulmonary fibrosis	rs3766823	-0.812680115	1.044786022	0.436661224
**GERD**	Idiopathic pulmonary fibrosis	rs3793577	-0.482233503	1.374881547	0.725779294
**GERD**	Idiopathic pulmonary fibrosis	rs3863241	1.226973684	0.901359728	0.173435485
**GERD**	Idiopathic pulmonary fibrosis	rs4300861	1.742616034	1.182855454	0.140689508
**GERD**	Idiopathic pulmonary fibrosis	rs4382592	-1.76744186	1.703392517	0.29945583
**GERD**	Idiopathic pulmonary fibrosis	rs4713692	-1.484848485	1.431810278	0.299715929
**GERD**	Idiopathic pulmonary fibrosis	rs569356	1.00295858	1.153674809	0.384650327
**GERD**	Idiopathic pulmonary fibrosis	rs6711584	-1.273109244	1.155744768	0.270657885
**GERD**	Idiopathic pulmonary fibrosis	rs7032155	0.46	1.391144737	0.740899103
**GERD**	Idiopathic pulmonary fibrosis	rs7241572	-0.144578313	1.375569063	0.916292996
**GERD**	Idiopathic pulmonary fibrosis	rs7527682	-1.119850187	1.022320074	0.273341101
**GERD**	Idiopathic pulmonary fibrosis	rs7541875	0.780730897	0.913844701	0.39291856
**GERD**	Idiopathic pulmonary fibrosis	rs7600261	-1.099526066	1.413521329	0.436649731
**GERD**	Idiopathic pulmonary fibrosis	rs7612999	0.869731801	1.199262705	0.468315795
**GERD**	Idiopathic pulmonary fibrosis	rs761777	0.255384615	0.950129156	0.788091775
**GERD**	Idiopathic pulmonary fibrosis	rs7675588	0.590659341	0.912026707	0.517221817
**GERD**	Idiopathic pulmonary fibrosis	rs7685686	-4.905982906	2.351002178	0.036909619
**GERD**	Idiopathic pulmonary fibrosis	rs773109	0.481967213	0.95714628	0.614580424
**GERD**	Idiopathic pulmonary fibrosis	rs7942368	-0.711191336	1.183256792	0.547809588
**GERD**	Idiopathic pulmonary fibrosis	rs903678	0.7578125	2.255999396	0.736938822
**GERD**	Idiopathic pulmonary fibrosis	rs9372625	-0.772277228	0.925203771	0.403880774
**GERD**	Idiopathic pulmonary fibrosis	rs9373363	-1.506802721	1.075405641	0.161169749
**GERD**	Idiopathic pulmonary fibrosis	rs942065	2.611872146	1.410009022	0.063971312
**GERD**	Idiopathic pulmonary fibrosis	rs9615905	-0.329004329	1.177080356	0.779854378
**GERD**	Idiopathic pulmonary fibrosis	rs9636202	-0.151234568	0.962819964	0.875186049
**GERD**	Idiopathic pulmonary fibrosis	rs9940128	-2.460526316	3.605438912	0.494955408
**GERD**	Idiopathic pulmonary fibrosis	All - Inverse variance weighted	0.359869692	0.157484747	0.022306482
**GERD**	Idiopathic pulmonary fibrosis	All - MR Egger	1.482156814	0.525761529	0.006646258
**GERD**	Chronic obstructive pulmonary disease	rs10010963	0.144133133	0.757855422	0.849163752
**GERD**	Chronic obstructive pulmonary disease	rs1011407	0.67717737	0.654730887	0.301003636
**GERD**	Chronic obstructive pulmonary disease	rs10133111	-0.183776536	0.399695531	0.645666013
**GERD**	Chronic obstructive pulmonary disease	rs1021363	0.635709804	0.522741176	0.223943727
**GERD**	Chronic obstructive pulmonary disease	rs10837002	1.734855469	0.494308594	0.000448679

**GERD**	Chronic obstructive pulmonary disease	rs11762636	-0.247772397	0.323133172	0.443211697
**GERD**	Chronic obstructive pulmonary disease	rs11953061	2.80497235	0.562497696	6.14E-07
**GERD**	Chronic obstructive pulmonary disease	rs12204714	0.930928571	0.629438776	0.139145146
**GERD**	Chronic obstructive pulmonary disease	rs12357321	1.658310502	0.593054795	0.005170451
**GERD**	Chronic obstructive pulmonary disease	rs12453010	0.291724675	0.317664935	0.358440466
**GERD**	Chronic obstructive pulmonary disease	rs12598916	0.87107764	0.420950311	0.038516794
**GERD**	Chronic obstructive pulmonary disease	rs12967855	1.180593625	0.534968127	0.027324643
**GERD**	Chronic obstructive pulmonary disease	rs12997558	-0.713372093	0.576265116	0.215744451
**GERD**	Chronic obstructive pulmonary disease	rs13107325	0.87752381	0.916456044	0.338305507
**GERD**	Chronic obstructive pulmonary disease	rs1334297	0.085132195	0.652585366	0.896207459
**GERD**	Chronic obstructive pulmonary disease	rs13409451	0.174673611	0.871298611	0.841109088
**GERD**	Chronic obstructive pulmonary disease	rs1431196	0.357725424	0.511555085	0.484370732
**GERD**	Chronic obstructive pulmonary disease	rs1479405	0.745492114	0.408498423	0.068007497
**GERD**	Chronic obstructive pulmonary disease	rs1510719	1.218286982	0.36647929	0.000886409
**GERD**	Chronic obstructive pulmonary disease	rs1592757	0.366718881	0.871314685	0.673842747
**GERD**	Chronic obstructive pulmonary disease	rs1596747	0.897733577	0.433890511	0.038543035
**GERD**	Chronic obstructive pulmonary disease	rs1716171	-1.05969434	0.550539623	0.054250821
**GERD**	Chronic obstructive pulmonary disease	rs17379561	0.635554324	0.314913525	0.043571809
**GERD**	Chronic obstructive pulmonary disease	rs1883842	0.92480212	0.537628975	0.085405301
**GERD**	Chronic obstructive pulmonary disease	rs1937450	1.019551084	0.371916409	0.006118827
**GERD**	Chronic obstructive pulmonary disease	rs2016933	0.6218327	0.528292776	0.239171253
**GERD**	Chronic obstructive pulmonary disease	rs2023878	0.828227273	0.324559809	0.010715328
**GERD**	Chronic obstructive pulmonary disease	rs2043539	0.142484181	0.694350282	0.837411928
**GERD**	Chronic obstructive pulmonary disease	rs215614	1.091183908	0.504800766	0.030648017
**GERD**	Chronic obstructive pulmonary disease	rs2164300	1.418592179	0.664145251	0.032682124
**GERD**	Chronic obstructive pulmonary disease	rs2240326	0.174509477	0.388964052	0.653682758
**GERD**	Chronic obstructive pulmonary disease	rs2396133	-0.21205188	0.449139098	0.636834259
**GERD**	Chronic obstructive pulmonary disease	rs2396766	0.499198697	0.388250814	0.198525638
**GERD**	Chronic obstructive pulmonary disease	rs2734839	1.1954	0.643178378	0.063086357
**GERD**	Chronic obstructive pulmonary disease	rs2782641	0.838993103	0.838165517	0.316832911
**GERD**	Chronic obstructive pulmonary disease	rs2834005	0.072642857	0.40562614	0.857868444
**GERD**	Chronic obstructive pulmonary disease	rs2838771	0.861665217	0.570126087	0.130696998
**GERD**	Chronic obstructive pulmonary disease	rs324769	1.946965318	0.702179191	0.00555853
**GERD**	Chronic obstructive pulmonary disease	rs329122	1.843581633	0.612382653	0.002608128
**GERD**	Chronic obstructive pulmonary disease	rs3766823	-0.035801441	0.41459366	0.931185742
**GERD**	Chronic obstructive pulmonary disease	rs3793577	0.398302538	0.605055838	0.510351451
**GERD**	Chronic obstructive pulmonary disease	rs3863241	0.199460197	0.396388158	0.614827786
**GERD**	Chronic obstructive pulmonary disease	rs4300861	0.8202827	0.506291139	0.105193649
**GERD**	Chronic obstructive pulmonary disease	rs4382592	1.691965116	0.776581395	0.029351355
**GERD**	Chronic obstructive pulmonary disease	rs4713692	-0.470481313	0.614742424	0.444074586
**GERD**	Chronic obstructive pulmonary disease	rs569356	0.549505917	0.51954142	0.290203684
**GERD**	Chronic obstructive pulmonary disease	rs6711584	0.542882353	0.500613445	0.278172316
**GERD**	Chronic obstructive pulmonary disease	rs6780459	-0.049421782	0.479613861	0.917927303
**GERD**	Chronic obstructive pulmonary disease	rs7032155	0.369059	0.61358	0.54751718
**GERD**	Chronic obstructive pulmonary disease	rs7206608	-0.337955172	0.428244828	0.430016472
**GERD**	Chronic obstructive pulmonary disease	rs7241572	0.601317269	0.582827309	0.302201153
**GERD**	Chronic obstructive pulmonary disease	rs7527682	-0.041099251	0.456565543	0.928272678

**GERD**	Chronic obstructive pulmonary disease	rs7541875	0.405089701	0.404627907	0.31675851
**GERD**	Chronic obstructive pulmonary disease	rs7600261	0.119096682	0.611943128	0.845690047
**GERD**	Chronic obstructive pulmonary disease	rs7612999	1.274532567	0.538555556	0.017953495
**GERD**	Chronic obstructive pulmonary disease	rs761777	0.276477231	0.410803077	0.500936822
**GERD**	Chronic obstructive pulmonary disease	rs7675588	0.734898352	0.43960989	0.094582373
**GERD**	Chronic obstructive pulmonary disease	rs7685686	3.310042735	1.017358974	0.00113967
**GERD**	Chronic obstructive pulmonary disease	rs773109	-0.478055738	0.423131148	0.25855835
**GERD**	Chronic obstructive pulmonary disease	rs7942368	-0.36868231	0.527151625	0.484311019
**GERD**	Chronic obstructive pulmonary disease	rs903678	2.597570313	1.018734375	0.010778431
**GERD**	Chronic obstructive pulmonary disease	rs903959	-0.206941532	0.492637097	0.674435108
**GERD**	Chronic obstructive pulmonary disease	rs9372625	0.129052475	0.421867987	0.759675267
**GERD**	Chronic obstructive pulmonary disease	rs9373363	0.923778912	0.428343537	0.031034434
**GERD**	Chronic obstructive pulmonary disease	rs942065	0.275149315	0.559557078	0.622912359
**GERD**	Chronic obstructive pulmonary disease	rs9542729	-0.058028727	0.485523636	0.904865122
**GERD**	Chronic obstructive pulmonary disease	rs9615905	0.204041991	0.516774892	0.692962982
**GERD**	Chronic obstructive pulmonary disease	rs9636202	0.855561728	0.43987037	0.051771187
**GERD**	Chronic obstructive pulmonary disease	rs9940128	0.378576316	1.573210526	0.80983467
**GERD**	Chronic obstructive pulmonary disease	All - Inverse variance weighted	0.499556774	0.079591212	3.46E-10
**GERD**	Chronic obstructive pulmonary disease	All - MR Egger	-0.065241218	0.275032483	0.813215564
**GERD**	Chronic bronchitis	rs10010963	0.215690361	2.819331325	0.939018065
**GERD**	Chronic bronchitis	rs1011407	6.83559633	2.568443425	0.007782176
**GERD**	Chronic bronchitis	rs10133111	0.920170391	1.490284916	0.536940619
**GERD**	Chronic bronchitis	rs1021363	-0.959090196	1.951278431	0.6230595
**GERD**	Chronic bronchitis	rs10837002	2.871535156	1.851625	0.120945076
**GERD**	Chronic bronchitis	rs11762636	2.040174334	1.20788862	0.091211425
**GERD**	Chronic bronchitis	rs11953061	-3.204580645	2.113313364	0.129423948
**GERD**	Chronic bronchitis	rs12204714	0.339076531	2.346234694	0.885090304
**GERD**	Chronic bronchitis	rs12357321	-1.236552511	2.219890411	0.577504791
**GERD**	Chronic bronchitis	rs12453010	-0.064213766	1.18578961	0.956813467
**GERD**	Chronic bronchitis	rs12598916	0.933913043	1.570341615	0.552030873
**GERD**	Chronic bronchitis	rs12967855	-0.656776892	1.996167331	0.74214136
**GERD**	Chronic bronchitis	rs12997558	-1.944269767	2.150544186	0.365951558
**GERD**	Chronic bronchitis	rs13107325	-3.805659341	3.392234432	0.261916051
**GERD**	Chronic bronchitis	rs1334297	-0.832117073	2.436682927	0.732730339
**GERD**	Chronic bronchitis	rs13409451	-0.718604167	3.258777778	0.825471338
**GERD**	Chronic bronchitis	rs1431196	3.235817797	1.909279661	0.090116287
**GERD**	Chronic bronchitis	rs1479405	1.160675079	1.520451104	0.445239504
**GERD**	Chronic bronchitis	rs1510719	0.540988166	1.361677515	0.691149665
**GERD**	Chronic bronchitis	rs1592757	2.125937063	3.255251748	0.51370525
**GERD**	Chronic bronchitis	rs1596747	0.184632847	1.618540146	0.909179378
**GERD**	Chronic bronchitis	rs1716171	-0.148414717	2.047592453	0.942217894
**GERD**	Chronic bronchitis	rs17379561	1.249749446	1.172144124	0.286329625
**GERD**	Chronic bronchitis	rs1883842	2.199424028	2.009378092	0.273700909
**GERD**	Chronic bronchitis	rs1937450	0.996795666	1.384928793	0.471681973
**GERD**	Chronic bronchitis	rs2016933	-0.456775665	1.969193916	0.816568512
**GERD**	Chronic bronchitis	rs2023878	-0.107474641	1.211763158	0.929326072
**GERD**	Chronic bronchitis	rs2043539	2.139214689	2.589344633	0.408712957

**GERD**	Chronic bronchitis	rs215614	0.864616858	1.876984674	0.6450559
**GERD**	Chronic bronchitis	rs2164300	2.513854749	2.478569832	0.310470155
**GERD**	Chronic bronchitis	rs2240326	0.075248039	1.449522876	0.958598588
**GERD**	Chronic bronchitis	rs2396133	1.116992481	1.674353383	0.504696305
**GERD**	Chronic bronchitis	rs2396766	3.143736156	1.447221498	0.029836329
**GERD**	Chronic bronchitis	rs2734839	0.976745946	2.404837838	0.684625956
**GERD**	Chronic bronchitis	rs2782641	-1.720186207	3.125441379	0.582057495
**GERD**	Chronic bronchitis	rs2834005	-0.683142857	1.510270517	0.65103022
**GERD**	Chronic bronchitis	rs2838771	-1.607186957	2.125682609	0.449600993
**GERD**	Chronic bronchitis	rs324769	2.462531792	2.627236994	0.348599848
**GERD**	Chronic bronchitis	rs329122	0.503817347	2.279423469	0.825070311
**GERD**	Chronic bronchitis	rs3766823	-0.111741787	1.552409222	0.942618147
**GERD**	Chronic bronchitis	rs3793577	0.090347716	2.259375635	0.968102763
**GERD**	Chronic bronchitis	rs3863241	-2.439575658	1.478575658	0.098953197
**GERD**	Chronic bronchitis	rs4300861	1.059253165	1.892447257	0.575665901
**GERD**	Chronic bronchitis	rs4382592	-2.153790698	2.899372093	0.457574142
**GERD**	Chronic bronchitis	rs4713692	-0.513545455	2.291454545	0.822669139
**GERD**	Chronic bronchitis	rs569356	-2.720352071	1.931378698	0.158982274
**GERD**	Chronic bronchitis	rs6711584	1.598726891	1.865063025	0.391335884
**GERD**	Chronic bronchitis	rs6780459	-0.841491749	1.790930693	0.638453028
**GERD**	Chronic bronchitis	rs7032155	2.025445	2.283175	0.375015525
**GERD**	Chronic bronchitis	rs7206608	-0.887996552	1.595503448	0.577826739
**GERD**	Chronic bronchitis	rs7241572	0.728674699	2.17526506	0.737638644
**GERD**	Chronic bronchitis	rs7527682	0.073043071	1.701269663	0.96575379
**GERD**	Chronic bronchitis	rs7541875	0.867518272	1.509498339	0.565489916
**GERD**	Chronic bronchitis	rs7600261	2.651796209	2.27835545	0.244461284
**GERD**	Chronic bronchitis	rs7612999	2.015183908	2.004444444	0.31472458
**GERD**	Chronic bronchitis	rs761777	1.564172308	1.532033846	0.30726503
**GERD**	Chronic bronchitis	rs7675588	1.366662088	1.646706044	0.406574468
**GERD**	Chronic bronchitis	rs7685686	-0.531588034	3.795196581	0.888605756
**GERD**	Chronic bronchitis	rs773109	0.855445902	1.578763934	0.587925045
**GERD**	Chronic bronchitis	rs7942368	3.044841155	1.96631769	0.12150218
**GERD**	Chronic bronchitis	rs903678	3.19596875	3.813796875	0.402029677
**GERD**	Chronic bronchitis	rs903959	-2.439923387	1.837233871	0.184164297
**GERD**	Chronic bronchitis	rs9372625	1.036141914	1.574557756	0.510504296
**GERD**	Chronic bronchitis	rs9373363	0.647156463	1.598911565	0.685662661
**GERD**	Chronic bronchitis	rs942065	0.23016895	2.083283105	0.912025729
**GERD**	Chronic bronchitis	rs9542729	4.766472727	1.826458182	0.009062676
**GERD**	Chronic bronchitis	rs9615905	-0.365565368	1.923424242	0.849262376
**GERD**	Chronic bronchitis	rs9636202	0.233526852	1.640546296	0.88680592
**GERD**	Chronic bronchitis	rs9940128	-5.423118421	5.871013158	0.355636893
**GERD**	Chronic bronchitis	All - Inverse variance weighted	0.574532787	0.220782904	0.009261373
**GERD**	Chronic bronchitis	All - MR Egger	1.079665936	0.782819208	0.172419057
**GERD**	Bronchiectasis	rs10010963	0.498938554	2.058210843	0.808459602
**GERD**	Bronchiectasis	rs1011407	0.276872171	1.777085627	0.876189681
**GERD**	Bronchiectasis	rs10133111	-0.068890223	1.086572626	0.949446874
**GERD**	Bronchiectasis	rs1021363	-2.2974	1.420282353	0.105756229

**GERD**	Bronchiectasis	rs10837002	-1.558164063	1.350105469	0.248456944
**GERD**	Bronchiectasis	rs11762636	-1.504094431	0.8751477	0.085673575
**GERD**	Bronchiectasis	rs11953061	-0.568018433	1.533769585	0.711128102
**GERD**	Bronchiectasis	rs12204714	1.981464286	1.705719388	0.245374004
**GERD**	Bronchiectasis	rs12357321	1.05673516	1.613844749	0.512600673
**GERD**	Bronchiectasis	rs12453010	-0.12916987	0.86345974	0.881083596
**GERD**	Bronchiectasis	rs12598916	0.508475155	1.142940994	0.656404222
**GERD**	Bronchiectasis	rs12967855	1.941952191	1.454669323	0.181883401
**GERD**	Bronchiectasis	rs12997558	0.122748837	1.562674419	0.937390171
**GERD**	Bronchiectasis	rs13107325	-2.93959707	2.550915751	0.249169339
**GERD**	Bronchiectasis	rs1334297	-1.813629268	1.76817561	0.305029936
**GERD**	Bronchiectasis	rs13409451	-1.863451389	2.362486111	0.430248124
**GERD**	Bronchiectasis	rs1431196	1.277101695	1.388016949	0.357525285
**GERD**	Bronchiectasis	rs1479405	-0.178281703	1.107946372	0.872162827
**GERD**	Bronchiectasis	rs1510719	-0.431215976	0.992195266	0.663847005
**GERD**	Bronchiectasis	rs1592757	0.482394406	2.364097902	0.838314351
**GERD**	Bronchiectasis	rs1596747	0.334566423	1.177656934	0.776337594
**GERD**	Bronchiectasis	rs1716171	-0.794143396	1.490411321	0.594147766
**GERD**	Bronchiectasis	rs17379561	1.675161863	0.85078714	0.048958222
**GERD**	Bronchiectasis	rs1883842	0.360134276	1.463819788	0.805663931
**GERD**	Bronchiectasis	rs1937450	-2.157733746	1.007083591	0.032148707
**GERD**	Bronchiectasis	rs2016933	3.052520913	1.436361217	0.033571899
**GERD**	Bronchiectasis	rs2023878	-0.377588517	0.877600478	0.667013039
**GERD**	Bronchiectasis	rs2043539	1.661966102	1.881502825	0.377064254
**GERD**	Bronchiectasis	rs215614	-0.35706092	1.374961686	0.795104523
**GERD**	Bronchiectasis	rs2164300	1.661832402	1.798234637	0.355410001
**GERD**	Bronchiectasis	rs2240326	-0.358117647	1.054124183	0.73405984
**GERD**	Bronchiectasis	rs2396133	-0.941236842	1.218041353	0.439672592
**GERD**	Bronchiectasis	rs2396766	-0.789159609	1.051491857	0.452945052
**GERD**	Bronchiectasis	rs2734839	-1.695189189	1.744827027	0.331273757
**GERD**	Bronchiectasis	rs2782641	-3.003951724	2.275055172	0.186706069
**GERD**	Bronchiectasis	rs2834005	-0.101665957	1.096085106	0.926099228
**GERD**	Bronchiectasis	rs2838771	1.756604348	1.544365217	0.255359379
**GERD**	Bronchiectasis	rs324769	3.110485549	1.910768786	0.103552191
**GERD**	Bronchiectasis	rs329122	-0.951877551	1.661561224	0.566724987
**GERD**	Bronchiectasis	rs3766823	-0.723213256	1.12207781	0.519231553
**GERD**	Bronchiectasis	rs3793577	-0.486114721	1.643720812	0.767428309
**GERD**	Bronchiectasis	rs3863241	0.420944079	1.075325658	0.695459245
**GERD**	Bronchiectasis	rs4300861	0.982848101	1.372054852	0.473785785
**GERD**	Bronchiectasis	rs4382592	0.334834302	2.107796512	0.873783008
**GERD**	Bronchiectasis	rs4713692	-0.42979798	1.665585859	0.796371308
**GERD**	Bronchiectasis	rs569356	1.400943787	1.41483432	0.322085062
**GERD**	Bronchiectasis	rs6711584	-1.085634454	1.358159664	0.424091964
**GERD**	Bronchiectasis	rs6780459	1.603419142	1.301132013	0.217827236
**GERD**	Bronchiectasis	rs7032155	0.083026	1.66387	0.96020261
**GERD**	Bronchiectasis	rs7206608	-1.038489655	1.159144828	0.370300735
**GERD**	Bronchiectasis	rs7241572	-0.473261044	1.582180723	0.764848898

**GERD**	Bronchiectasis	rs7527682	0.011046105	1.242441948	0.992906389
**GERD**	Bronchiectasis	rs7541875	-1.042302326	1.098152824	0.342548693
**GERD**	Bronchiectasis	rs7600261	-1.825232227	1.662995261	0.272397942
**GERD**	Bronchiectasis	rs7612999	1.177088123	1.467371648	0.422452023
**GERD**	Bronchiectasis	rs761777	0.782206154	1.112892308	0.482143748
**GERD**	Bronchiectasis	rs7675588	0.808604396	1.190494505	0.497000224
**GERD**	Bronchiectasis	rs7685686	3.909128205	2.770632479	0.158269542
**GERD**	Bronchiectasis	rs773109	-0.673183607	1.150295082	0.55839517
**GERD**	Bronchiectasis	rs7942368	0.883176895	1.429956679	0.536822606
**GERD**	Bronchiectasis	rs903678	2.379453125	2.763265625	0.389181954
**GERD**	Bronchiectasis	rs903959	-0.036952742	1.336939516	0.977949437
**GERD**	Bronchiectasis	rs9372625	-1.019537954	1.142636964	0.37224951
**GERD**	Bronchiectasis	rs9373363	1.150503401	1.158979592	0.320862753
**GERD**	Bronchiectasis	rs942065	-2.031114155	1.51483105	0.179979247
**GERD**	Bronchiectasis	rs9542729	0.791058182	1.315145455	0.547507928
**GERD**	Bronchiectasis	rs9615905	-0.467350649	1.400952381	0.738685224
**GERD**	Bronchiectasis	rs9636202	-0.573694444	1.195719136	0.63137651
**GERD**	Bronchiectasis	rs9940128	-7.388684211	4.270131579	0.083573531
**GERD**	Bronchiectasis	All - Inverse variance weighted	-0.073932883	0.160493976	0.645043771
**GERD**	Bronchiectasis	All - MR Egger	-0.156080028	0.569032754	0.784705658

Table S10. Results of all Mendelian Randomization analyses estimates for associations between GERD and risk of chronic respiratory diseases from UKB GROUP.

outcome	exposure	method	nsnp	SE	OR	95% CI		P-value
**Asthma**	GERD	MR Egger	71	0.023	0.99	0.95	1.04	0.666
**Asthma**	GERD	Weighted median	71	0.006	1.03	1.02	1.04	<0.001
**Asthma**	GERD	Inverse variance weighted	71	0.007	1.03	1.02	1.04	<0.001
**Asthma**	GERD	Simple mode	71	0.017	1.04	1.01	1.08	0.017
**Asthma**	GERD	Weighted mode	71	0.013	1.04	1.01	1.06	0.008
***Idiopathic pulmonary fibrosis**	GERD	MR Egger	58	0.53	4.40	1.57	12.34	0.007
***Idiopathic pulmonary fibrosis**	GERD	Weighted median	58	0.21	1.27	0.84	1.93	0.255
***Idiopathic pulmonary fibrosis**	GERD	Inverse variance weighted	58	0.16	1.43	1.05	1.95	0.022
***Idiopathic pulmonary fibrosis**	GERD	Simple mode	58	0.45	1.09	0.45	2.66	0.843
***Idiopathic pulmonary fibrosis**	GERD	Weighted mode	58	0.43	1.20	0.52	2.78	0.666
**Chronic obstructive pulmonary disease**	GERD	MR Egger	58	0.003	0.996	0.991	1.001	0.149
**Chronic obstructive pulmonary disease**	GERD	Weighted median	58	0.001	1.003	1.001	1.006	0.001
**Chronic obstructive pulmonary disease**	GERD	Inverse variance weighted	58	0.001	1.004	1.003	1.006	<0.001
**Chronic obstructive pulmonary disease**	GERD	Simple mode	58	0.002	1.004	1.000	1.008	0.047
**Chronic obstructive pulmonary disease**	GERD	Weighted mode	58	0.002	1.003	1.000	1.007	0.060
**Chronic bronchitis**	GERD	MR Egger	71	0.005	1.004	0.994	1.015	0.396
**Chronic bronchitis**	GERD	Weighted median	71	0.002	1.010	1.006	1.014	<0.001
**Chronic bronchitis**	GERD	Inverse variance weighted	71	0.001	1.011	1.008	1.014	<0.001
**Chronic bronchitis**	GERD	Simple mode	71	0.005	1.016	1.007	1.026	0.001
**Chronic bronchitis**	GERD	Weighted mode	71	0.004	1.010	1.002	1.019	0.017
**Bronchiectasis**	GERD	MR Egger	42	0.002	0.998	0.993	1.003	0.389
**Bronchiectasis**	GERD	Weighted median	42	0.001	1.000	0.998	1.002	0.945
**Bronchiectasis**	GERD	Inverse variance weighted	42	0.001	1.001	0.999	1.002	0.285
**Bronchiectasis**	GERD	Simple mode	42	0.002	1.000	0.996	1.004	0.854
**Bronchiectasis**	GERD	Weighted mode	42	0.002	1.000	0.996	1.003	0.841

***：As there is currently no publicly available summary data on IPF from the UKB working group, we utilized the original source of data. OR: Odds ratio; CI: Confidence interval. SE: Stand error.**

Table S11. Results of all Mendelian Randomization analyses estimates for associations between GERD and risk of chronic respiratory diseases from FinnGen GROUP.

outcome	exposure	method	nsnp	SE	OR	95% CI		P-value
**Asthma**	GERD	MR Egger	69	0.22	0.70	0.46	1.06	0.097
**Asthma**	GERD	Weighted median	69	0.07	1.37	1.19	1.58	<0.001
**Asthma**	GERD	Inverse variance weighted	69	0.07	1.67	1.46	1.91	<0.001
**Asthma**	GERD	Simple mode	69	0.15	1.33	1.00	1.78	0.058
**Asthma**	GERD	Weighted mode	69	0.12	1.24	0.99	1.55	0.071
**Idiopathic pulmonary fibrosis**	GERD	MR Egger	69	0.80	0.42	0.09	2.04	0.288
**Idiopathic pulmonary fibrosis**	GERD	Weighted median	69	0.27	0.95	0.56	1.61	0.852
**Idiopathic pulmonary fibrosis**	GERD	Inverse variance weighted	69	0.23	1.02	0.66	1.59	0.919
**Idiopathic pulmonary fibrosis**	GERD	Simple mode	69	0.62	0.96	0.28	3.26	0.952
**Idiopathic pulmonary fibrosis**	GERD	Weighted mode	69	0.54	0.81	0.28	2.33	0.692
**Chronic obstructive pulmonary disease**	GERD	MR Egger	69	0.28	0.94	0.55	1.61	0.813
**Chronic obstructive pulmonary disease**	GERD	Weighted median	69	0.09	1.44	1.21	1.73	<0.001
**Chronic obstructive pulmonary disease**	GERD	Inverse variance weighted	69	0.08	1.65	1.41	1.93	<0.001
**Chronic obstructive pulmonary disease**	GERD	Simple mode	69	0.24	1.33	0.84	2.13	0.229
**Chronic obstructive pulmonary disease**	GERD	Weighted mode	69	0.20	1.28	0.85	1.90	0.237
**Chronic bronchitis**	GERD	MR Egger	69	0.78	2.94	0.63	13.65	0.172
**Chronic bronchitis**	GERD	Weighted median	69	0.32	1.84	0.99	3.42	0.053
**Chronic bronchitis**	GERD	Inverse variance weighted	69	0.22	1.78	1.15	2.74	0.009
**Chronic bronchitis**	GERD	Simple mode	69	0.64	1.60	0.46	5.63	0.465
**Chronic bronchitis**	GERD	Weighted mode	69	0.58	1.82	0.58	5.68	0.309
**Bronchiectasis**	GERD	MR Egger	69	0.57	0.86	0.28	2.61	0.785
**Bronchiectasis**	GERD	Weighted median	69	0.23	0.85	0.54	1.34	0.484
**Bronchiectasis**	GERD	Inverse variance weighted	69	0.16	0.93	0.68	1.27	0.645
**Bronchiectasis**	GERD	Simple mode	69	0.49	0.75	0.28	1.98	0.565
**Bronchiectasis**	GERD	Weighted mode	69	0.46	0.71	0.29	1.76	0.461

OR: Odds ratio; CI: Confidence interval. SE: Stand error.
